# BMP10 functions independently from BMP9 for the development of a proper arteriovenous network

**DOI:** 10.1007/s10456-022-09859-0

**Published:** 2022-11-08

**Authors:** Hyunwoo Choi, Bo-Gyeong Kim, Yong Hwan Kim, Se-Jin Lee, Young Jae Lee, S. Paul Oh

**Affiliations:** 1 Barrow Aneurysm & AVM Research Center, Department of Translational Neuroscience, Barrow Neurological Institute, 350 W Thomas Road, Phoenix, AZ 85013 USA; 2 Lee Gil Ya Cancer and Diabetes Institute, Gachon University, 155 Gaetbeol-Ro, Yeonsu-Gu, 21999 Incheon, Republic of Korea; 3 The Jackson Laboratory for Genomic Medicine, Farmington, CT USA; 4 Department of Genetics and Genome Sciences, University of Connecticut School of Medicine, Farmington, CT USA; 5 Department of Biochemistry, Gachon University College of Medicine, Incheon, Republic of Korea; 6 Department of Physiology and Functional Genomics, University of Florida College of Medicine, Gainesville, FL USA

**Keywords:** Bone morphogenetic protein 9, Bone morphogenetic protein 10, Endoglin, Arteriovenous malformation, Hereditary hemorrhagic telangiectasia

## Abstract

Hereditary hemorrhagic telangiectasia (HHT) is a genetic vascular disorder characterized by the presence of arteriovenous malformation (AVM) in multiple organs. HHT is caused by mutations in genes encoding major constituents for transforming growth factor-β (TGF-β) family signaling: endoglin (ENG), activin receptor-like kinase 1 (ALK1), and SMAD4. The identity of physiological ligands for this ENG-ALK1 signaling pertinent to AVM formation has yet to be clearly determined. To investigate whether bone morphogenetic protein 9 (BMP9), BMP10, or both are physiological ligands of ENG-ALK1 signaling involved in arteriovenous network formation, we generated a novel *Bmp10* conditional knockout mouse strain. We examined whether global *Bmp10*-inducible knockout (iKO) mice develop AVMs at neonatal and adult stages in comparison with control, *Bmp9*-KO, and *Bmp9/10*-double KO (dKO) mice. *Bmp10*-iKO and *Bmp9/10*-dKO mice showed AVMs in developing retina, postnatal brain, and adult wounded skin, while *Bmp9*-KO did not display any noticeable vascular defects. *Bmp10* deficiency resulted in increased proliferation and size of endothelial cells in AVM vessels. The impaired neurovascular integrity in the brain and retina of *Bmp10*-iKO and *Bmp9/10*-dKO mice was detected. *Bmp9/10*-dKO mice exhibited the lethality and vascular malformation similar to *Bmp10*-iKO mice, but their phenotypes were more pronounced. Administration of BMP10 protein, but not BMP9 protein, prevented retinal AVM in *Bmp9/10*-dKO and endothelial-specific *Eng*-iKO mice. These data indicate that BMP10 is indispensable for the development of a proper arteriovenous network, whereas BMP9 has limited compensatory functions for the loss of BMP10. We suggest that BMP10 is the most relevant physiological ligand of the ENG-ALK1 signaling pathway pertinent to HHT pathogenesis.

**Supplementary Information:**

The online version contains supplementary material available at 10.1007/s10456-022-09859-0.

## Introduction

Hereditary hemorrhagic telangiectasia (HHT) is a genetic vascular disorder characterized by the presence of arteriovenous malformation (AVM) in multiple organs [1–3]. AVMs are direct shunts between arteries and veins without intervening capillary beds. AVMs are prone to rupture which cause gushing bleeding. Rupture of small AVMs in the mucosal surface leads to frequent epistaxis and gastrointestinal tract bleeding and results in anemia. Larger AVMs in the liver and lungs additionally cause life-threatening conditions such as liver failure, embolic stroke, brain abscess, and heart failure. Brain AVMs occurring in about 10% of HHT patients can result in devastating conditions such as neurological damages, stroke, and death.

All three identified genes associated with HHT are constituents of signal transduction of TGF-β family members (ENG: type III receptor; ALK1: type I receptor; SMAD4: intracellular mediator) [4–6]; thus, HHT has been considered a disease caused by defects in the signaling of a TGF-β family member(s). Due to promiscuous interactions of ENG and ALK1 with a number of TGF-β superfamily ligands, the identity of the ligand of this signal transduction pertinent to HHT pathogenesis remains unclear. Defining the authentic ligand(s) for the ENG-ALK1 signaling pertinent to HHT pathogenesis would greatly facilitate the process of identifying downstream targets and enhance our knowledge of the pathogenetic mechanisms of disease.

Based on crystal structure and binding assays, bone morphogenetic protein 9 (BMP9) was predicted as a potential ligand of ALK1 [7, 8]. Since then numerous biochemical and cellular experiments have shown that BMP9 can signal through ALK1 in endothelial cells and regulate various angiogenic properties [7, 9, 10]. BMP9 polymorphic sites were shown to be associated with patients with HHT-like vascular disorder [11]. Structural studies have identified specific amino acid residues in the extracellular domain of ENG as a binding site for BMP9 [12], and the mutations in the site were associated with HHT [13]. A recent study identified a missense BMP9 variant in a family with HHT [14].

BMP10, the closest family member of BMP9, has biochemical properties similar to BMP9 in regulating ALK1 downstream signals, such as SMAD1/5 phosphorylation and *Id1* expression [7]. *Bmp10*-null mice were shown to die at embryonic day 10.5 with phenotypes remarkably similar to *Alk1*-null embryos, including AV shunts between the dorsal aorta and cardinal vein [15]. Similarly, simultaneous knockdown of both *bmp10* and *bmp10*-like in zebrafish phenocopied *acvrl1* mutants [16]. Recently, it was shown that *bmp10*-null adult fish exhibited some HHT-like phenotypes [17].

There is mounting evidence showing functional overlaps between BMP9 and BMP10. Replacing BMP10 with BMP9 could partially overcome mid-gestational vascular phenotypes of *Bmp10*-null embryos [18]. BMP10 neutralizing antibody treatment showed angiogenic defects in retinal vascular development only on *Bmp9*-null background [18, 19]. Treatment with both BMP9 and BMP10 blocking antibodies, but not with individual antibodies, in neonates resulted in AVM formation in the developing retina [19–23]. These results suggest that BMP9 and BMP10 are both relevant physiological ligands for ENG-ALK1 in the process of proper formation of arteriovenous networks. However, it remains to be determined if deficiency of BMP9, BMP10, or both BMP9 and BMP10 is the major cause of AVM development. In addition, it is important to determine whether BMP9 and BMP10 have the equal therapeutic potential for HHT.

*Alk1-*, *Eng-* or *Smad4-*inducible knockout (iKO) mice serve as HHT models as they exhibit AVM phenotypes in multiple organs and tissues at neonatal and adult stages [24–27]. *Bmp9*-null and *Bmp10*-iKO mice have been reported but their vascular phenotypes relevant to AVMs have not been carefully and systemically examined [28–30]. In this paper, we introduce a novel *Bmp10*-conditional allele and define the unique and overlapping roles of these two ligands in angiogenesis, arteriovenous network formation, and HHT pathogenesis by examining vascular phenotypes of *Bmp9*-null, *Bmp10*-iKO, and *Bmp9/10*-double KO mice.

## Materials and methods

### Mice

*Bmp10* mutant mice bearing conditional knockout (*Bmp10*^2loxP(2f)^) or lacZ reporter (*Bmp10*^lacZ(3f)^) alleles were generated at Gachon University. Generation of *Bmp9*-knockout mice (*Bmp9*-KO) on a C57BL/6J background was previously described [19]. ROSA26^CreER/+^ (R26^CreER/+^) was utilized for inducible global gene deletion ([31]; JAX stock #008463). *Tagln*-Cre [32], where Cre recombinase is transiently expressed in cardiomyocytes at a mid-gestational period ([33]; JAX stock #017491), was used to delete *Bmp10* in embryonic cardiomyocytes, and *Scl*-CreER mice were used for endothelial cell-specific deletion of *Eng* [34]. To induce Cre activity for deletion of the *Bmp10* gene, tamoxifen (Sigma, T5648) was given to control (*Bmp10*^2f/2f^), *Bmp9*-KO (*Bmp9*^−/−^;*Bmp10*^2f/2f^), *Bmp10*-iKO (R26^CreER/+^;*Bmp10*^2f/2f^), and *Bmp9/10*-dKO (R26^CreER/+^;*Bmp9*^−/−^;*Bmp10*^2f/2f^) mice with following dosages and schedules: for postnatal day (P) 6 analysis and survival study, 50 μg tamoxifen dissolved in corn oil (Sigma, C8267) was intragastrically injected at P1, P2, P3, and P4; for neonatal AVM analysis, tamoxifen (75 μg/g body weight for control and *Bmp10*-iKO littermates, 35 μg/g body weight for *Bmp9*-KO and *Bmp9/10*-dKO littermates) was intragastrically injected at P2 and P3; for wound-induced AVM study and survival study at adult mice, tamoxifen (100 μg/g body weight) was intraperitoneally injected for three consecutive days. All mice were maintained in a C57BL/6J × 129Sv hybrid background to compare their phenotypes. We utilized CreER-positive *Bmp10*-iKO and CreER-negative littermate control mice derived from intercross of R26^CreER/+^;*Bmp10*^2f/2f^ with *Bmp10*^2f/2f^. CreER-positive *Bmp9/10*-dKO (R26^CreER/+^;*Bmp9*^−/−^;*Bmp10*^2f/2f^) and CreER-negative littermate R26^+/+^;*Bmp9*^−/−^;*Bmp10*^2f/2f^ (*Bmp9*-KO) mice were derived from the intercross of R26^CreER/+^;*Bmp9*^−/−^;*Bmp10*^2f/2f^ and *Bmp9*^−/−^;*Bmp10*^2f/2f^ mice. Neonatal *Scl*-CreER;*Eng*^2f/2f^ and control littermates were given an intragastric injection of 50 μg tamoxifen at P1, P2, and P3, and the retinas were harvested at P7. Tamoxifen-injected CreER-negative littermates were used as controls. For generation of *Bmp10*^1f^ allele, tamoxifen treated R26^CreER/+^;*Bmp10*^2f/+^ male mice were mated with WT mice. Among the offspring, a R26^+/+^;*Bmp10*^1f/+^ mouse was selected. Transmission of *Bmp10*-1f allele and absence of the 2f allele were confirmed for more than eight generations. All housing, breeding and procedures were performed according to the NIH Guide for the Care and Use of Experimental Animals and approved by the Institutional Animal Care and Use Committees of Gachon University (LCDI-2017–0069 and LCDI-2018–0086), University of Florida (UF 20,141,417), and Barrow Neurological Institute (530).

### Construction of conditional knockout vector for targeting Bmp10

A BAC DNA [129S7/AB2.2 library, bMQ438f03 (87,389,727–87,451,271)] containing mouse *Bmp10* gene was obtained from Source BioScience (UK). A vector targeted *Bmp10* was constructed by using a homologous recombination method [35]. The mouse *Bmp10* gene contains two exons (Online Figure IA). An 18.6-kb genomic DNA fragment of *Bmp10* was retrieved from the BAC DNA and inserted into pLMJ235 possessing the diphtheria toxin A fragment (DT-A) gene as a negative selection marker. A frt-SD/SA-IRES-LacZ-Neo-frt-loxP (LacZ/Neo) cassette including a reporter (LacZ) and a positive selection marker (Neo, neomycin-resistant gene) was inserted at 477-bp upstream of exon 2. Downstream *loxP* sequence was inserted at 234-bp downstream of exon 2.

### Generation for Bmp10 conditional knockout mice

The linearized targeting vector (45 μg) was electroporated into 2 × 10^7^ J1 embryonic stem (ES) cells. Approximately 300 G418-resistant colonies were randomly picked. Homologous recombination was screened by genomic Southern blot analyses using external 5′-probes (Online Figure IB) and tested the first loxP site by PCR analysis (Online Figure IC) using specific primers (Online Table I). Targeted ES cells were injected into blastocysts of the C57BL/6 (B6) strain. ES cell culture and blastocyst injections were performed by standard methods. Male chimeras were bred to B6 females to establish the *Bmp10*^lacZ(3f)^ strain on a 129/B6 hybrid background. Germ-line transmission of the targeted allele was confirmed by genotyping (Online Figure IC). *Bmp10*^lacZ/+^ mice were crossed with FLP–deleter mice (ROSA26^FLPe^) to remove the LacZ/Neo cassette flanked by the frt sequences for generating the conditional *Bmp10*^2f/+^ mice. Genotypes of wild-type, *Bmp10*^2f/+^ and *Bmp10*^1f/+^, alleles were analyzed by PCR (Online Figure ID and 1E) using the primer sets listed in Online Table I.

### Whole-mount X-gal staining

Whole-mount X-gal staining was performed using littermate control and *Bmp10*^3f/+^ mice carrying a lacZ reporter. Fresh tissues were fixed with fixation solution [1% formaldehyde, 0.2% glutaraldehyde, 2 mM MgCl_2_, 5 mM EGTA, and 0.02% NP-40 in PBS (pH7.4)] for 10 min with gentle agitation. After three times washing with PBS, the samples were incubated in X-gal staining solution [5 mM K_3_[Fe(CN)_6_], 5 mM K_4_[Fe(CN)_6_], 2 mM MgCl_2_, 0.01% Na-deoxycholate, 0.02% NP-40, and 0.75 mg/mL X-gal in PBS (pH7.4)] at 37 °C overnight with gentle agitation. After three times washing with PBS, samples were sequentially dehydrated using increasing concentration of methanol, cleared with a BABB organic solvent (benzyl alcohol/benzyl benzoate = 1:1; Fisher, AC148390010, AC105860010) and photographed under a stereomicroscope with a CCD camera (Leica). For the histological analysis, X-gal-stained samples were embedded in paraffin following hydration with ethanol and clearing with xylene. The sections with 7 μm thickness were counterstained with nuclear fast red (Vector laboratories).

### Histology

Collected tissues were fixed in 4% paraformaldehyde, dehydrated with the serial dilutions of ethanol, cleared with xylene, and embedded in paraffin. Seven-μm-thick sections were rehydrated with ethanol and stained with hematoxylin and eosin.

### Antibodies

For immunostaining: anti-CD31 (Biocare medical, CM303B; 1:200), anti-Ki67 (Thermo, RM9106S1; 1:200), anti-VE-Cadherin (BioLegend, 348,501; 1:100), anti-Erg1/2/3-Alexa Fluor 647 (Santa Cruz, sc-376293; 1:100), and anti-SMA-Cy3 (Sigma, C6198; 1:200) antibodies were used. For western blotting: anti-BMP10-GFD (R&D systems, MAB6038; 1:1000), anti-BMP10-Pro (R&D systems, AF3956; 1:1500), and anti-TNNI3 (abcam, ab56357; 1:3000) antibodies were used.

### Immunofluorescence

The eyeballs of neonatal pups were enucleated and fixed in 4% paraformaldehyde for 50 min at room temperature. Retinas were dissected, blocked for 1 h in blocking buffer [0.25% Triton X-100, 0.01% Na-deoxycholate, 3% BSA in PBS (pH 7.4)], and then incubated in blocking buffer at 4 °C overnight with Isolectin B4 (IB4; Sigma, L2140) or primary antibodies as described in antibodies. The retinas were washed five times with PBST (0.25% Triton X-100 in PBS) and incubated with appropriate fluorescence-conjugated secondary antibodies (Invitrogen) in blocking buffer at RT for 2 h. The retinas were washed five times with PBST and mounted on microscope glass slides with ProLong™ Gold Antifade Mountant (Invitrogen, P36930). The images were acquired by fluorescence microscopy (Keyence, BZ-X710).

### Morphometric analysis

Morphometric measurements of retinal vasculature were done using ImageJ software. The radial length of the retina vessel was measured as the distance from the optic disk to the outermost vessel in the vascular front. Two measurements were obtained from each retina and averaged. Vascular density was measured as CD31-positive vessel area in 500 μm × 500 μm fields located between an artery and a vein in each retina and presented as a percentage. The diameter of arteries and veins was measured at 500 μm from the optic disk. The number of branch points was measured manually in four 500 µm × 500 µm fields located between an artery and a vein in each retina and averaged. The number of tip cells was measured in 300 µm × 500 µm fields located at the peripheral front between an artery and a vein. Endothelial cell size in veins was quantified as total cell area with CD31 and VE-Cadherin-stained boundaries divided by the number of Erg1/2/3-positive endothelial cell nuclei. Endothelial cell number was calculated as the total number of Erg1/2/3-positive endothelial cell nuclei per 100 μm length of vessels.

### Western blot analysis

Mouse tissues were homogenized and prepared in ice-cold lysis buffer [50 mM Tris–HCl (pH 7.4), 150 mM NaCl, 1 mM EDTA, 1% Triton X-100, 0.5% SDS, 0.5% Na-deoxycholate, phosphatase inhibitor mixture (Sigma, P5726 and P0044), and complete protease inhibitor mixture (Thermo, 87786)]. The supernatants after centrifugation at 15,000 × g, 4 °C for 30 min were used for measuring the protein concentration by DC Protein Assay kit (Bio-rad, 5000116). Samples were separated using SDS–polyacrylamide gels and transferred onto nitrocellulose membranes. The membranes were blocked with 5% non-fat milk in TBST (Tris-buffered saline with 0.2% Tween-20) for 1 h, probed using primary antibodies as described in antibodies, and further incubated with appropriate HRP-conjugated secondary antibodies. The bands were visualized by ECL substrate (Pierce, PI34096), and band intensities were quantified with Image Lab (Bio-rad).

### Reverse transcription-quantitative real-time polymerase chain reaction (RT-qPCR)

RNAs were purified from various tissues of mice using a NucleoSpin RNA kit (Macherey–Nagel) and then reverse-transcribed using SuperScript III (Invitrogen). Comparative ΔC_T_ method was applied to identify relative changes in mRNA expression using SYBR Premix (Applied biosystems, 4,367,659) and ABI StepOne real-time system (Applied biosystems) with the corresponding primer sets: *mBmp10* (#1: 5′-ACA TCA TCC GGA GCT TCA AGA ACG-3′, 5′-AAC CGC AGT TCA GCC ATG ACG-3′; #2: 5′-GCA GAT GAG GTC GAA CAT GA-3′, 5′-GGC CTG AAT AAT TGC GTG TT-3′; #3: 5′-GGA TCC ACC AGA GTA CAT GCT-3′, 5′-GTC CAC GCC ATC ATA CAT CA-3′), *Actin* (5′-CCT GAA CCC TAA GGC CAA CCG-3′, 5′-GCT CAT AGC TCT TCT CCA GGG-3′). The expression levels were normalized to *Actin* as indicated.

### Enzyme-linked immunosorbent assay (ELISA)

BMP9 and BMP10 protein levels in plasma or serum were measured using BMP9/10 ELISA kits (R&D systems, DY5566, DY2926) according to the manufacturer’s instructions. Briefly, a 96-well microtiter plate was coated with capture antibody and incubated with blocking solution [1% bovine serum albumin in PBS (pH 7.4)]. 100 μl of samples was added to each well and incubated at 4 °C overnight. After washing the plate five times with PBST (0.05% Tween-20 in PBS), the biotinylated detection antibody was incubated for 2 h at RT. The color change was detected via HRP-conjugated streptoavidin antibody. Neonatal and adult samples were measured with 1/4 or 1/8 dilution and with 1 or 1/2 dilution, respectively.

### Diffusible iodine-based contrast-enhanced computed tomography (diceCT)

P6 pups were fixed in 10% formalin for 2 days, washed with PBS, and incubated in Lugol’s iodine solution (1.25% iodine and 2.5% potassium iodine in distilled water; Carolina Biological Supply) for 6 days. The samples were scanned in a Phoenix v|tome|x M scanner (GE Measurement & control). The resulting 2D X-ray data were processed using datos|x software v2.3 and analyzed using VG StudioMax 3.0 (Volume Graphics).

### Skin wound generation and blue latex dye perfusion assay

Six to 16-week-old male and female mice were deeply anesthetized by placing the animal within an induction chamber and introducing 4% isoflurane gas, and anesthesia was maintained by 2% isoflurane. One wound was inflicted on the mid-dorsum and the other on the untagged ear using a sterile 4 mm biopsy punch (Integra, 33–34). Betadine was applied to the wound. To delete conditional alleles of *Bmp10*, tamoxifen at 100 μg/g body weight was intraperitoneally injected for three consecutive days from the day of wounding (day 0). To examine arterial vasculature and AVMs, the perfusion of latex blue dye was performed at day 6 for *Bmp9/10*-dKO mice and at day 7 for control, *Bmp9*-KO and *Bmp10*-iKO mice. Latex dye (Connecticut valley biological supply company) was injected into the left ventricle with a 26-gauge syringe, after sequential perfusion with 10 ml of dilator (10 unit/ml heparin, 0.04 mg/ml papaverine, and 100 μM sodium nitroprusside) and 10 ml of 10% formalin. Injected mice were briefly washed in PBS, and hair on the back was removed. After overnight post-fixation in 10% formalin, the dorsal skin and ear were collected, dehydrated, and cleared with a BABB organic solvent. For neonates, blue latex dye was injected with a 27.5-gauge syringe after anesthesia with ice and perfusion with 4 ml of the dilator and 4 ml of 10% formalin. The images were acquired by a stereomicroscope with a CCD camera.

### Embryo isolation and whole-mount staining

Pregnant mice were euthanized by cervical dislocation following anesthesia with isoflurane. Embryos were fixed in 4% paraformaldehyde at 4 °C overnight, washed with PBS, blocked for 1 h at room temperature in blocking buffer [0.3% BSA and 0.1% Tween-20 in PBS (pH 7.4)], and then incubated with primary antibodies in blocking buffer at 4 °C overnight. Embryos were washed five times with PBST (0.1% Tween-20 in PBS) and further incubated with appropriate fluorescence-conjugated secondary antibodies (Invitrogen) in blocking buffer at 4 °C overnight. Embryos were washed with PBST, dehydrated with serial dilution of methanol (25%, 50%, 75%, 90%, and 100% methanol in PBS), cleared with a BABB organic solvent, and photographed by fluorescence microscopy (Keyence, BZ-X710).

### Complete blood count and hemoglobin level measurement

Blood was obtained from the tail vein of adults or superficial temporal vein of pups and collected into tubes containing EDTA (Sarstedt Inc, NC9414041; RAM Scientific, 07–6011; Greiner Bio-One, 450470). A complete blood count was carried out using Element HT5 (Heska). Hemoglobin level was examined using a hemoglobin photometer (Stanbio Laboratory, Hemopoint H2).

### Tracer injection experiment

For Alexa Fluor 555 cadaverine tracer injection and detection in postnatal mice at P8, 40 μl of Alexa Fluor 555 cadaverine tracer (1 mg/ml, Invitrogen) was injected intraperitoneally. After 2 h, mice were anesthetized using ice and then perfused intracardially with 6 ml of the dilator and 4% paraformaldehyde. After dissection of eyes and brains, eyes were fixed in 4% paraformaldehyde for an additional 1 h at room temperature. Retinas were isolated and mounted on microscope glass slides with ProLong™ Gold Antifade Mountant. Brains were post-fixed at 4 °C overnight and skulls were removed. For tracer leakage detection, the images of whole brains and retinas were acquired using a fluorescence microscope (Keyence, BZ-X710). Leakage was quantified by measuring the fluorescence intensity of the brain and retina images with ImageJ.

### Supplementation with BMP9 and BMP10

*Bmp9/10*-dKO mice were injected intragastrically with 50 μg tamoxifen dissolved in corn oil (Sigma, C8267) at P1, P2, P3, and P4. Control PBS, 100 ng of mouse BMP9 protein (R&D systems, 5566-BP), or 100 ng of mouse BMP10 protein (R&D systems, 6038-BP) was injected intraperitoneally and daily on the opposite side of the milk spot until the sample collection. Proteins were reconstituted in 4 mM HCl containing 0.1% BSA (R&D systems, Reconstitution buffer 04) according to the manufacturer’s instructions. 100 ng of protein was diluted with 4 μl PBS right before the injection and injected using a Hamilton syringe (Model 701 RN SYR). The analysis was performed at P8. Neonatal *Scl*-CreER;*Eng*^2f/2f^ and control littermates were given intragastric injections of 50 μg tamoxifen at P1, P2, and P3, and the retinas were harvested at P7.

### Statistical analysis

Data are expressed as mean ± standard error of the mean (SEM) or mean ± standard deviation of the mean (SD) with at least three independent experiments. Statistical analyses were performed using GraphPad Prism 8. The data distribution was assessed with D’Agostino & Pearson normality test or Shapiro–Wilk normality test. For normally distributed data, statistical comparisons between two groups and among multiple groups were performed by unpaired two-tailed student’s *t* test and either one-way or two-way analysis of variance (ANOVA) followed by Tukey’s post hoc test, respectively. For non-normally distributed data, statistical comparisons were performed by Kruskal–Wallis test followed by Dunn’s post hoc test. Survival curves were created by the method of Kaplan–Meier and were compared using the log-rank test. Assessments with *P* < 0.05 were considered statistically significant.

## Results

### Generation of mice with *Bmp10* conditional knockout and lacZ reporter alleles

To investigate the role of BMP10 in neonatal and adult stages by circumventing the early embryonic lethality of conventional *Bmp10* knockout mice and to systemically examine *Bmp10* expression patterns during development, we generated conditional (*Bmp10*^2loxP(2f)^) and lacZ reporter (*Bmp10*^lacZ(3f)^) strains (Fig. S1a). To visualize *Bmp10* expression patterns, lacZ expression in *Bmp10*^lacZ/+^ and littermate control embryos and neonates at various stages was analyzed by X-gal staining. *Bmp10* expression began to be found in the developing hearts and liver bud at E8.5 and became enriched in ventricles and right atrium at E14.5 (Fig. S2a). Interestingly, cardiac *Bmp10* expression was found not only in the right atrium but also in the ventricles, in contrast to a previous report that detected *Bmp10* transcripts only in both atria at E18.5 by in situ hybridization [15]. The expression persisted at least until postnatal day (P)7 in the ventricular trabeculae and became restricted to the right atrium afterward (Fig. S2a). Histological sections also revealed that the X-gal-positive cells were detected mainly in cardiac trabeculae and liver (Fig. S2b), but not in the spleen, brain, lung, and thymus (Fig. S2c).

To confirm the functionality of the *Bmp10* inducible allele (*Bmp10*^2f^), we generated a null allele (*Bmp10*^1f^) from the floxed allele. Homozygous embryos for the *Bmp10*^1f^ allele recapitulated the phenotype of previously reported *Bmp10*-null embryos, exhibiting lethal phenotypes with growth-retardation, pericardial effusion, and the lack of mature yolk sac vasculature at E9.5 (Fig. S3a) [15]. Homozygous embryos for the *Bmp10*^lacZ(3f)^ allele exhibited essentially the same phenotype as *Bmp10*^1f/1f^, indicating that *Bmp10*^lacZ(3f)^ is likely a null allele (Fig. S3b). To examine the importance of BMP10 expressed in cardiomyocytes, *Bmp10*^2f^ mice were crossed with *Tagln*-Cre mice, where Cre recombinase is transiently expressed in cardiomyocytes during embryonic stages from E8.5 [33]. No live *Tagln*-Cre;*Bmp10*^2f/2f^ mice was found at the weaning stage. Timed mating studies showed that *Tagln*-Cre;*Bmp10*^2f/2f^ embryos showed lethality at E9.5 with a gross morphology essentially the same as that of *Bmp10*^1f/1f^ mice (Fig. S3c). Whole-mount immunofluorescent staining with anti-CD31 antibodies exhibited disorganized and immature blood vessels in the cranial, trunk, and intersomitic vessels (Fig. S3d). These results demonstrate that cardiac BMP10 plays a crucial and independent role on vessel development and survival.

### Global depletion of neonatal *Bmp10* leads to retarded outgrowth, hypervascularization, and increased tip cell number in the retina vasculature

**Fig. 1:**
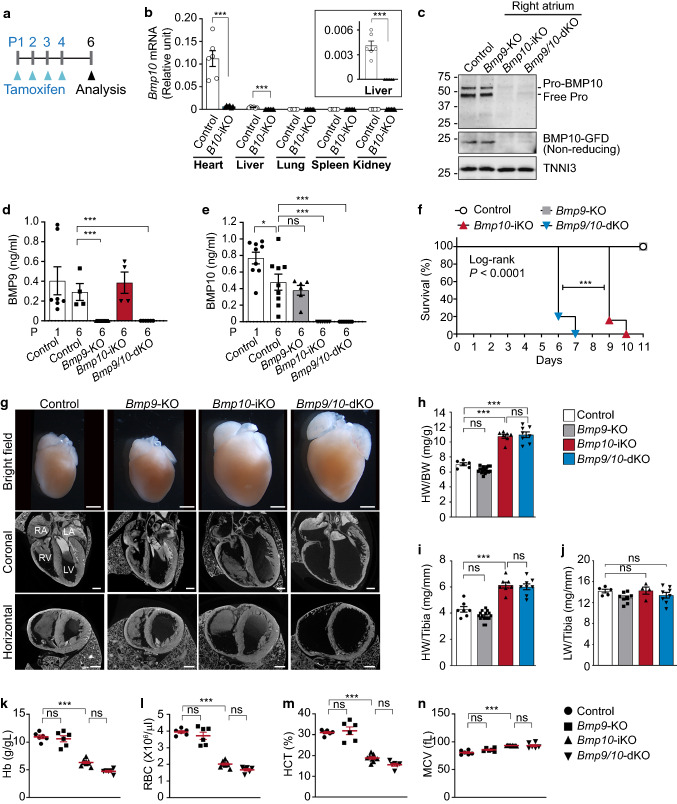
*Bmp10*-inducible null mutants die prematurely showing enlarged hearts. **a** Schematic representation of the experimental strategy for *Bmp10* global deletion in control (CreER-negative *Bmp10*-iKO), *Bmp9*-KO (CreER-negative *Bmp9/10*-dKO), *Bmp10*-inducible KO (*Bmp10*-iKO), and *Bmp9/10*-double KO (*Bmp9/10*-dKO) mice using R26^CreER/+^ line. Arrowheads indicate intragastric injections of 50 μg tamoxifen at P1, P2, P3, and P4 and the analysis at P6. **b** Levels of *Bmp10* mRNA were detected by RT-qPCR in the hearts, livers, lungs, spleens, and kidneys from tamoxifen-injected P6 littermate control and *Bmp10*-iKO mice. Inset indicates the level of *Bmp10* mRNA in the livers. The mRNA levels are normalized to *Actin*. Data are mean ± SEM. Unpaired *t* test (*n* = 6 mice per group). **c** Representative immunoblots show the levels of BMP10 proteins in the right atrium at P6. Unprocessed monomer (Pro-BMP10) and free prodomain (Free Pro) of BMP10 proteins were determined with anti-BMP10-Pro antibodies. Dimer of growth factor domain (GFD) was blotted using anti-BMP10-GFD antibodies under the non-reducing condition. TNNI3 was used as a loading control. Numbers indicate the molecular mass in kDa. **d** and **e**, Plasmatic levels of BMP9 (**d**) and BMP10 (**e**) proteins assessed by ELISA in control, *Bmp9*-KO, *Bmp10*-iKO, and *Bmp9/10*-dKO mice at P1 and P6 as indicated. Data are mean ± SEM. One-way ANOVA followed by Tukey’s post hoc test (*n* = 4 to 9 mice per group). **f** Survival curves for control (*n* = 15 mice), *Bmp9*-KO (*n* = 15), *Bmp10*-iKO (*n* = 19), and *Bmp9/10*-dKO mice (*n* = 19) generated by the Kaplan–Meier method. Log-rank test (Match SPSS and SAS) was performed to determine significance. *P* < 0.0001. To compare the *Bmp10*-iKO and *Bmp9/10*-dKO survival curves, Mantel-Cox log-rank test was performed. g, Gross morphology (*upper*), and coronal (*middle*) and horizontal (*lower*) slices from 3D diceCT scanned images of control, *Bmp9*-KO, *Bmp10*-iKO, and *Bmp9/10*-dKO P6 hearts. *RA* right atrium, *LA* left atrium, *RV* right ventricle, *LV* left ventricle. Scale bars, 1 mm. **h**–**j** Quantification of heart weight/body weight (HW/BW, **h**), heart weight/tibia length (HW/Tibia, **i**), and lung weight/tibia length (LW/Tibia, **j**) ratios. Data are mean ± SEM. One-way ANOVA followed by Tukey’s post hoc test (*n* = 6 to 16 mice per group). **k**–**n**, Scatter plots measuring hemoglobin level (Hb, **k**), red blood cell count (RBC, **l**), hematocrit (HCT, **m**), and mean corpuscle volume (MCV, **n**) to assess anemia. Data represent individual mice and mean ± SEM. One-way ANOVA followed by Tukey’s post hoc test (*n* = 6 to 9 mice per group)

To investigate overlapping as well as independent roles of BMP9 and BMP10 in postnatal life, we characterized R26^CreER/+^;*Bmp10*^2f/2f^ (*Bmp10*-iKO) and R26^CreER/+^;*Bmp9*^−/−^;*Bmp10*-iKO (*Bmp9/10*-dKO) mice and compared their phenotypes with those in CreER-negative littermate control (R26^+/+^;*Bmp10*^2f/2f^), *Bmp9*-KO (R26^+/+^;*Bmp9*^−/−^;*Bmp10*^2f/2f^) mice. Global *Bmp10* deletion was induced by four consecutive days of intragastric injections of 50 μg tamoxifen starting from P1 (Fig. 1a). RT-qPCR analysis showed almost undetectable levels of *Bmp10* transcripts in the liver, atria, and ventricles of *Bmp10*-iKO mice compared to littermate control mice (Fig. 1b). Western blotting analysis revealed that BMP10 proteins, unprocessed monomer (Pro-BMP10), free prodomain (Pro), and growth factor domain (GFD) dimers were detected in right atrium samples of control and *Bmp9*-KO, but undetectable in those of *Bmp10*-iKO and *Bmp9/10*-dKO mice (Fig. 1c). Circulating BMP9 and BMP10 protein levels in plasma were examined by ELISA. BMP9 and BMP10 protein levels were not detected in *Bmp9*-KO and *Bmp10*-iKO mice at P6, respectively. There was no difference in the plasma BMP9 level between control and *Bmp10*-iKO P6 pups. Likewise, no difference was found in the plasma BMP10 level between control and *Bmp9*-KO P6 pups (Fig. 1d, e).

*Bmp9/10*-dKO and *Bmp10*-iKO mice resulted in lethality by P7 and P10, respectively, while no lethality was found in littermate control and *Bmp9*-KO mice (Fig. 1f). This result indicates that BMP10 is essential for survival and that BMP9 can partially compensate for BMP10 loss in terms of viability. Up on autopsy, it was noticeable that the hearts were greatly enlarged in both *Bmp10*-iKO and *Bmp9/10*-dKO mice at P6 (Fig. 1g). Diffusible iodine-based contrast-enhanced computed tomography (DiceCT) images showed that control and *Bmp9*-KO mice revealed no apparent abnormalities in heart morphology. However, *Bmp10*-iKO and *Bmp9/10*-dKO mice had the enlarged heart phenotype, along with anatomic features of ventricular dilatation and thinning of the myocardial wall (Fig. 1g, and Fig. S4b). Accordingly, the heart weight/body weight (HW/BW) ratio and heart weight/tibia length (HW/Tibia) were significantly increased, whereas the lung weight/tibia length (LW/Tibia) was not affected in *Bmp10*-iKO and *Bmp9/10*-dKO mice (Fig. 1h–j). Complete blood count exhibited significant reductions in hemoglobin levels, red blood cell (RBC) counts, and hematocrit levels, indicating anemia in *Bmp10*-iKO and *Bmp9/10*-dKO mice (Fig. 1k–n). It has been previously reported that a high-dose and prolonged tamoxifen treatment on R26^CreER^ mice causes anemia and premature death [36]. To exclude the possibility that the anemia and premature death phenotype in *Bmp10*-iKO and *Bmp9/10*-dKO mice are related to the direct hematological toxicity caused by the systemic activation of R26^CreER/+^, we compared the complete blood counts in neonatal R26^CreER/+^;*Bmp10*^+/+^ and their littermate controls (R26^+/+^;*Bmp10*^+/+^ or R26^+/+^;*Bmp10*^2f/+^) after treatment with the same tamoxifen regimen (Fig. S5a). We found no statistically significant differences in hemoglobin, red blood cell count, and hematocrit levels in R26^CreER/+^ mice compared to controls (Fig. S5b–e).

**Fig. 2:**
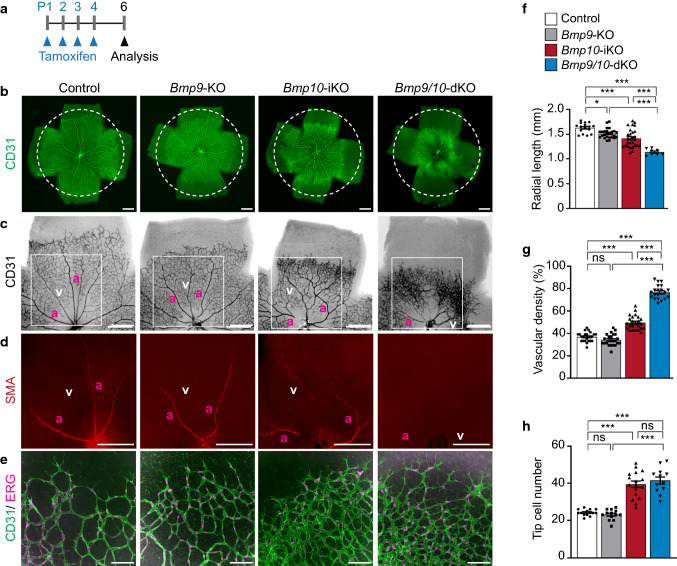
Postnatal *Bmp10* deletion leads to delayed progression of retinal vessels. **a** Schematic representation of the experimental strategy for *Bmp10* global deletion in control, *Bmp9*-KO, *Bmp10*-iKO, and *Bmp9/10*-dKO mice. Arrowheads indicate intragastric injections of 50 μg tamoxifen at P1, P2, P3, and P4 and the analysis at P6. **b–e** CD31, smooth muscle actin (SMA), and ERG1/2/3 staining of whole-mount P6 retinas from control, *Bmp9*-KO, *Bmp10*-iKO, and *Bmp9/10*-dKO mice. Dotted circles represent outgrowth of the control retina (**b**). Scale bars, 500 μm (**b–e**); 100 m (**e**). *a* artery, *v* vein. **f–h** Quantification of the vessel radial length (**f**, *n* = 8 to 16 retinas per group), vessel density in the vascular front (**g**, *n* = 20 to 24 areas from 5 to 6 retinas per group), and total tip cell number (**h**, *n* = 12 to 17 of areas from 5 to 7 retinas per group). Data are mean ± SEM. One-way analysis of variance (ANOVA) followed by Tukey’s post hoc test

We also examined the role of BMP9 and BMP10 in the development of retinal vasculature. P6 retinas immunostained with an endothelial marker (CD31) and smooth muscle alpha-actin (SMA) showed normal vascular development with artery-specific SMA staining in control and *Bmp9*-KO retinas (Fig. 2a–e). *Bmp10*-iKO and *Bmp9/10*-dKO retinas, however, exhibited abnormal vascular development, such as significantly reduced vessel outgrowth (Fig. 2b,c,f), and increased vascular density (Fig. 2b, g) and tip cell number (Fig. 2d, h) at the periphery. Radial expansion and hypervascularization phenotypes were more pronounced in *Bmp9/10*-dKO retinas compared with *Bmp10*-iKO retinas (Fig. 2f, g). It is worthwhile to note that these phenotypes in *Bmp9/10*-dKO are remarkably similar to *Bmp9*-null retinas treated with BMP10 neutral antibodies [18, 19]. Strong SMA-positive staining was found mostly in arteries of control and *Bmp9*-null retinas, but it was detected in some veins of *Bmp10*-iKO retina (Fig. 2d). Interestingly, no detectable SMA staining was found in both arteries and veins of *Bmp9/10*-dKO retina (Fig. 2d), perhaps due to severe defects in the maturation of vessels during angiogenesis. Both analyses of retinal vasculature and survival data suggest that BMP9 can partially compensate for the loss of BMP10.

### *Bmp10* deletion alone results in retinal AVMs with increases in proliferation and size of endothelial cells

**Fig. 3:**
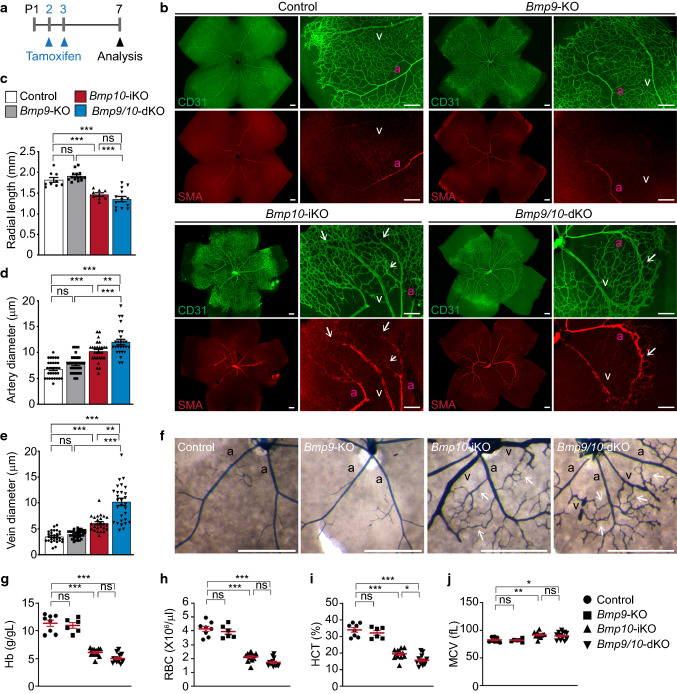
*Bmp10* deficiency leads to the development of arteriovenous malformation in neonatal retinas. **a** Schematic representation of *Bmp10* deletion. Arrowheads indicate intragastric tamoxifen injections at P2 and P3 and the analysis at P7. **b** CD31 and SMA staining of control, *Bmp9*-KO, *Bmp10*-iKO, and *Bmp9/10*-dKO P7 retinas. *a* artery, *v* vein. Scale bars, 200 μm. **c–e** Quantification of the vessel radial length (**c**, *n* = 8 to 14 retinas per group), artery diameter (**d**, *n* = 26 to 31 arteries from 6 to 8 retinas per group), and vein diameter (**e**, *n* = 25 to 34 veins from 6 to 8 retinas per group). Data are mean ± SEM. One-way ANOVA followed by Tukey’s post hoc test. **f** Representative images of the latex dye-perfused retinas of control, *Bmp9*-KO, *Bmp10*-iKO, and *Bmp9/10*-dKO mice at P7. Arrows mark arteriovenous shunts. *a* artery, *v* vein. Scale bars, 500 μm. **g–j** Scatter plots measuring Hb (**g**), RBC (**h**), HCT (**i**), and MCV (**j**) to assess hemorrhage. Data represent individual mice and mean ± SEM. One-way ANOVA followed by Tukey’s post hoc test (*n* = 6 to 13 mice per group)

To examine AVM phenotypes in *Bmp9* and *Bmp10* single and double knockout mutants, we had to change the tamoxifen injection regimen, because four consecutive days of injection starting from P1 resulted in too strong general angiogenesis defects to observe AVM phenotypes (Fig. 2b–d). After trying various regimens, we decided to change the tamoxifen regimen to 2 days of injection at P2 and P3 (Fig. 3a). This condition extended the lethality of *Bmp9/10*-dKO mice to P8 and allowed us to harvest the samples at P7, whereas vascular expansion and vessel morphology of *Bmp9*-KO retinas were indistinguishable from those of control retinas, both *Bmp10*-iKO and *Bmp9/10*-dKO retinas showed a marked reduction in vascular outgrowth (Fig. 3b, c) and increased diameters of both arteries and veins compared to control (Fig. 3d, e). Compared to artery-specific SMA staining in control and *Bmp9*-KO retinas, abnormal SMA staining was detected in the venous branches of *Bmp10*-iKO and *Bmp9/10*-dKO. CD31-stained vessels where SMA-positive enlarged venous vessels are present appeared to be arteriovenous (AV) shunts. To confirm these are indeed AV shuts, latex dye was perfused through the left ventricle of the heart. Latex dye does not pass through the capillaries, and thus, it is mostly retained in the arterial branches of a normal vascular network but it crosses to venous branches through AV shunts. We found that the latex dye was confined in the arteries of control and *Bmp9*-KO retinas, but it was detected in the venous branches and AV shunts in *Bmp10*-iKO and *Bmp9/10*-dKO retinas (Fig. 3f). Vessel enlargement in *Bmp9/10*-dKO appeared to be more apparent than that in *Bmp10*-iKO. Anemia phenotypes were observed in both *Bmp10*-iKO and *Bmp9/10*-dKO mice (Fig. 3g–j). These data demonstrate that BMP10 has an independent function from BMP9 for the development of a proper arteriovenous network.

**Fig. 4:**
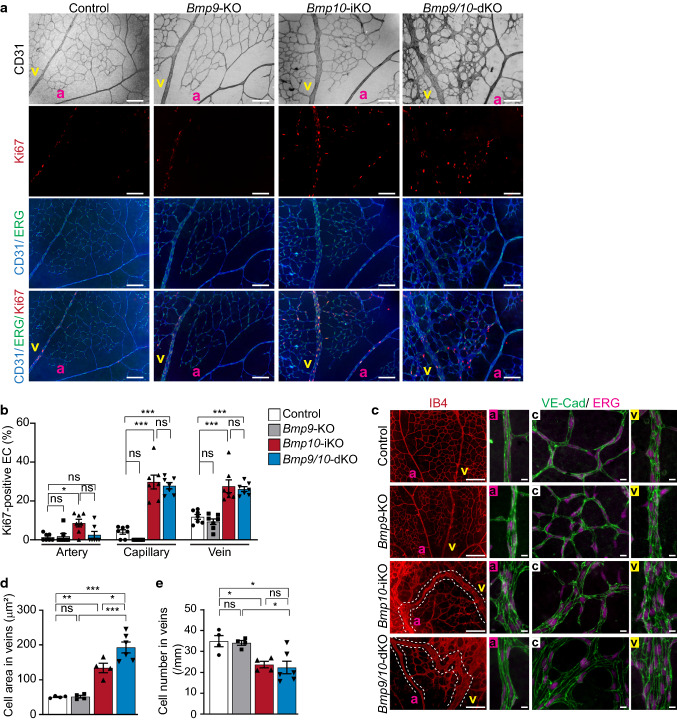
Proliferation and size of endothelial cells are increased in *Bmp10*-deleted retinas. **a** CD31, Ki67, and ERG1/2/3 staining in vascular plexus of control, *Bmp9*-KO, *Bmp10*-iKO, and *Bmp9/10*-dKO P7 retinas. *a* artery, *v* vein. Scale bars, 100 μm. **b** Quantification of the number of Ki67 and ERG1/2/3 double positive nuclei of endothelial cells per vascular area involved in AVM. Data are mean ± SEM. One-way ANOVA followed by Tukey’s post hoc test (*n* = 7 retinas per group). **c** IB4, VE-Cad, and ERG1/2/3 staining of control, *Bmp9*-KO, *Bmp10*-iKO, and *Bmp9/10*-dKO P7 retinas. Higher magnified images of vessels in the artery (a), capillary (c), and vein (v) are shown. The arteriovenous shunts are outlined by dotted lines. Scale bars, 100 μm; 10 μm (magnified images in a, c, and v). **d** and **e** Quantification of areas (**d**) and numbers (**e**) of endothelial cells in veins. Data are mean ± SEM. One-way ANOVA followed by Tukey’s post hoc test (*n* = 4 to 6 retinas per group)

To examine whether abnormal vasculature in *Bmp10*-iKO and *Bmp9/10*-dKO retinas is associated with an increase in EC proliferation, a characteristic of AVM shown in *Alk1*-, *Eng*- or *Smad4*-deleted retina [24–26, 37, 38], we immunolabeled retinas with antibodies against Ki67, a proliferation-associating protein, and ERG, an EC-specific nuclear marker (Fig. 4a). The number of proliferating ECs, Ki67/ERG-double positive cells, was increased more than twofold in all types of vessels associated with AVMs in *Bmp10*-iKO and *Bmp9/10*-dKO (Fig. 4b). The percentage of proliferating EC in *Bmp10*-iKO was similar to that in *Bmp9/10*-dKO (Fig. 4b).

Deletion of *Eng* or *Smad4* also increased endothelial cell size, which might contribute to increased vessel diameter and vascular malformations [26, 38, 39]. To examine whether *Bmp10* deletion could change cell size, we assessed individual endothelial cell area with VE-Cad and ERG staining (Fig. 4c). As shown in Fig. 3d and e, diameters in all types of vessels were increased in AVMs of *Bmp10*-iKO and *Bmp9/10*-dKO. Compared to the corresponding vessels in control and *Bmp9*-KO retinas, the size of venous ECs increased 2.5 and fourfold in *Bmp10*-iKO and *Bmp9/10*-dKO, respectively (Fig. 4d), while cell numbers in a given area were lower in the veins of these mutants (Fig. 4e). In addition, multiple endothelial cell nuclei cover the walls of AV-connecting capillaries in AVMs, while a single endothelial cell constructs the capillary walls in the controls (Fig. 4c). Taken together, the alteration of endothelial cell properties such as increased proliferation and cell size is associated with the AVM development caused by *Bmp10* loss.

### *Bmp10* deletion develops brain AVM and disrupts blood–brain barrier integrity

**Fig. 5:**
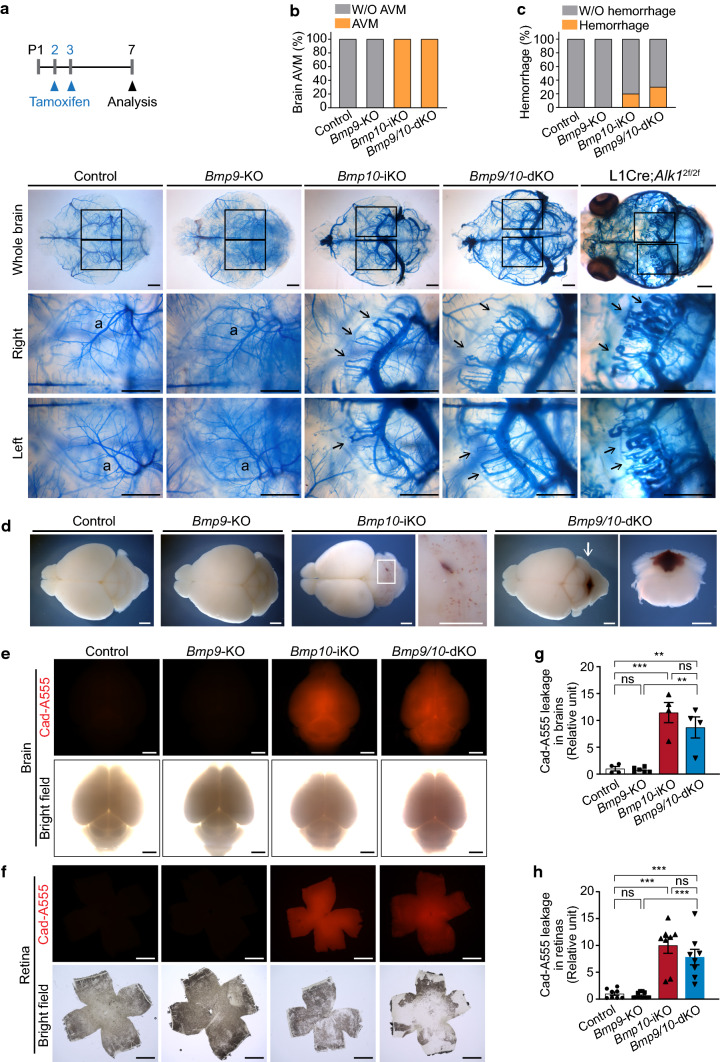
*Bmp10* deletion results in brain AVMs and disrupts blood–brain barrier integrity in neonatal mice. **a** Schematic representation of *Bmp10* deletion in neonatal mice. Arrowheads indicate intragastric tamoxifen injections at P2 and P3 and the analysis at P7 (*upper*). Representative images of the latex dye-injected cerebrovasculature in the brains of control, *Bmp9*-KO, *Bmp10*-iKO, *Bmp9/10*-dKO, and L1Cre-*Alk1* mice (*lower*). Magnified images of the boxes are shown in right and left hemispheres. Arrows mark arteriovenous shunts. a, artery; v, vein. Scale bars, 1 mm. **b** Percentage of brain AVMs of control (*n* = 4 mice), *Bmp9*-KO (*n* = 7), *Bmp10*-iKO (*n* = 10), and *Bmp9/10*-dKO mice (*n* = 10). **c** Percentage of visible bleeding in brains. **d** Representative images of the brains showing internal visible hemorrhage after the perfusion. A magnified image of the box in the *Bmp10*-iKO brain is shown in right panel. An arrow in the *Bmp9/10*-dKO brain indicates coronally dissected position showing internal bleeding and the image of coronal dissected brain slice is shown in right panel. Scale bars, 1 mm. **e** and **f** Fluorescent whole brain (**e**) and retina (**f**) images injected with Alexa Fluor 555-cadaverine (Cad-A555) at P8. Scale bars, 1 mm. **g** and **h**, Quantification of fluorescence intensity in brains (**g**, *n* = 4 to 6 mice per group) and retinas (**h**, *n* = 8 to 12 retinas per group). Data are mean ± SEM. One-way ANOVA followed by Tukey’s post hoc test

We also investigated whether *Bmp10* deletion develops AVM in the brain because deletion of *Alk1*, *Eng*, or *Smad4* gene resulted in brain AVMs [27, 40–42]. To visualize the arteriovenous shuts, latex dye was perfused through the left ventricle of the heart. The latex dye was mostly retained in the arterial branches of control and *Bmp9*-KO brains (Fig. 5a). However, in *Bmp10*-deleted brains, latex dye was found in veins as well as arterial branches, indicating the presence of arteriovenous shunts. Higher magnified views revealed multiple AV shunts consisting of enlarged arteries and veins with thicker apertures on the side of the venous connection. These AV fistulae were remarkably similar to those in the *Alk1*-, *Eng*-, or *Smad4*-deleted brain [27, 40, 41]. *Bmp10* deletion induced brain AVMs with 100% penetration in the tested *Bmp10*-iKO (*n* = 10) and *Bmp9/10*-dKO (*n* = 10) brains (Fig. 5b). Twenty to thirty percent of *Bmp10*-deleted brains displayed hemorrhagic signs (Fig. 5c, d). Microhemorrhage is a common symptom of brain AVMs [43].

In the central nervous system, blood vessels are the central component of the neurovascular unit that regulates the blood–brain barrier (BBB) and blood-retina barrier. We tested whether these barriers are impaired in *Bmp10*-iKO and *Bmp9/10*-dKO brains and retinas. Extravasation of the BBB-impermeable fluorescent tracer Alexa Fluor 555-cadaverine was observed in postnatal *Bmp10*-iKO and *Bmp9/10*-dKO brains, indicating compromised BBB integrity (Fig. 5e, g). Similar extravasation was observed in *Bmp10*-iKO and *Bmp9/10*-dKO retinas, indicating that the blood-retinal barrier was also impaired in these mutant mice (Fig. 5f, h).

### *Bmp10* deficiency develops de novo wound-induced skin AVMs in adult stages

**Fig. 6:**
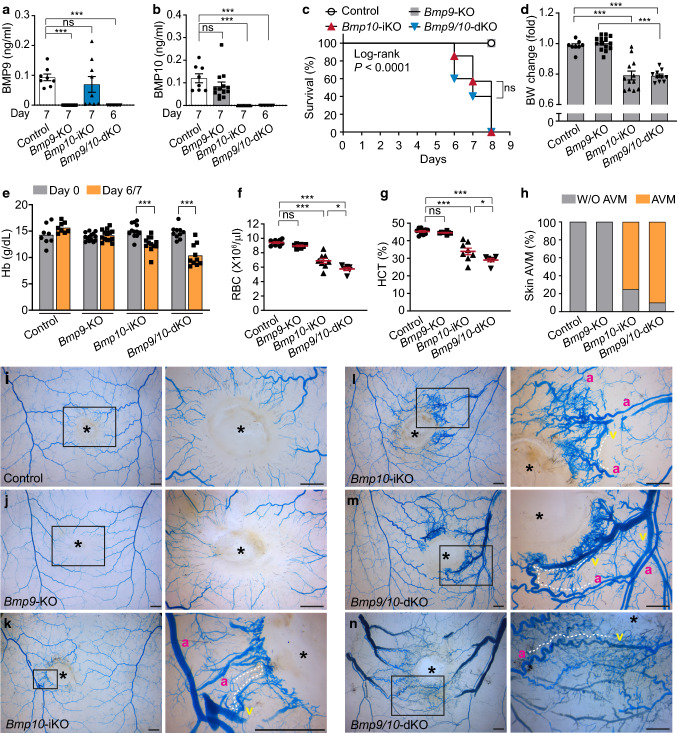
*Bmp10* deletion induces de novo AVM formation in response to skin wounding in adult mice. **a** and **b** *Bmp10* gene deletion was induced by intraperitoneal injection of tamoxifen at 100 μg/g body weight for three consecutive days from the day of wounding (day 0) in 6 ~ 8-week-old adult mice. Skin vasculatures were examined by latex dye injection at day 7 (control, *Bmp9*-KO, and *Bmp10*-iKO) or at day 6 (*Bmp9/10*-dKO). Plasmatic levels of BMP9 (**a**) and BMP10 (**b**) proteins in control, *Bmp9*-KO, *Bmp10*-iKO, and *Bmp9/10*-dKO mice were assessed by ELISA at Day 6 or Day 7 as indicated. Data are mean ± SEM. Kruskal–Wallis test followed by Dunn’s post hoc test (*n* = 8 to 13 mice per group). **c** Survival curves for control (*n* = 9 mice), *Bmp9*-KO (*n* = 9), *Bmp10*-iKO (*n* = 7), and *Bmp9/10*-dKO mice (*n* = 5) generated by the Kaplan–Meier method. Log-rank test (Match SPSS and SAS) was performed to determine significance. *P* < 0.0001. To compare the *Bmp10*-iKO and *Bmp9/10*-dKO survival curves, Mantel-Cox log-rank test was performed. **d** Percentile change of body weight from Day 0. Data are mean ± SEM. One-way ANOVA followed by Tukey’s post hoc test (*n* = 8 to 14 mice per group). **e** Hemoglobin levels in the beginning and end of the study period. Data are mean ± SEM. Two-way ANOVA followed by Tukey’s post hoc test (*n* = 8 to 14 mice per group). **f** and **g** Scatter plots measuring RBC (**f**) and HCT (**g**) at the end day of the study. Data represent mean ± SEM. One-way ANOVA followed by Tukey’s post hoc test (*n* = 6 to 10 mice per group). **h** Percentage of skin wound-induced AVMs (*n* = 8 to 14 mice per group). **i–n** Latex dye-injected vasculature surrounding the wound in the dorsal skin of control (**i**), *Bmp9*-KO (**j**), *Bmp10*-iKO (**k**, **l**), and *Bmp9/10*-dKO (**m**, **n**) mice. The magnified images of boxed area in the left panels are shown in the right panels. The wound sites are indicated by asterisks. Some arteriovenous shunts are outlined by dotted lines as examples. a artery, v vein. Scale bars, 1 mm

To investigate the role of BMP9 and BMP10 in adult stages, we induced deletion of *Bmp10* by 3 consecutive days of tamoxifen injection to R26^CreER/+^;*Bmp10*^2f/2f^ and R26^CreER/+^;*Bmp9*^−/−^;*Bmp10*^2f/2f^ adult mice. BMP9 or BMP10 depletion was confirmed by measuring protein levels in sera using ELISA (Fig. 6a, b). *Bmp9/10*-dKO and *Bmp10*-iKO mice reached to the moribund stage by day 8 post-injection (Fig. 6c). This result demonstrates that BMP10 is indispensable for adult life. These mutant mice exhibited weight loss (Fig. 6d) and a moderate level of anemia (Fig. 6e–g), but the lethality in *Bmp9/10*-dKO is likely due to defects in the gastrointestinal tract as these mutant mice showed diarrhea, starting from days 5–6. To exclude the possibility that the anemia phenotype is caused by the hematological defects seen in R26^CreER^ mice [36], adult R26^CreER/+^ and R26^+/+^ (*Bmp10*^+/+^ or *Bmp10*^2f/+^) mice were treated with the same tamoxifen regimen (3 days injections; 100 μg/g bw). We did not find any differences between these two groups (Fig. S5f–j), indicating that the anemia phenotype in *Bmp10*-iKO and *Bmp9/10*-dKO mice is unlikely derived from the Cre toxicity of R26^CreER^ mice.

It was previously demonstrated that deficiency of *Alk1*, *Eng*, or *Smad4* in adult stages develops de novo AVMs in subdermal vessels in response to wounds [27, 40, 44]. We compared the wound-induced skin AVM phenotype in *Bmp9*, *Bmp10*-iKOs, and *Bmp9/10*-dKOs with that observed in the mutant mice deficient in an HHT-causing gene. Subdermal blood vessels were visualized with latex dye perfusion either at day 7 for control, *Bmp9*-KO, and *Bmp10*-iKO mice or at day 6 for *Bmp9/10*-dKO mice due to their lethality from day 7. Over 80% of *Bmp10*-depleted mice developed subdermal vessel AVM in skins and ears (Fig. 6h, S6a). In wound areas of control mice, latex dye stayed only in arteries (Fig. 6i). Similar to the controls, no obvious vascular abnormality was found in *Bmp9*-KO mice (Fig. 6j). However, *Bmp10*-iKO as well as *Bmp9/10*-dKO mice showed abnormal artery-vein connections at various degrees near the wounds (Fig. 6k–n). As shown in skin AVM data, the wound-induced AVM phenotype was observed in the wounded ears of *Bmp10*-iKO and *Bmp9/10*-dKO mice, but not in the ears of control and *Bmp9*-KO mice (Fig. S6b). These data demonstrate that BMP10 is essential for survival and has a non-overlapping function in the development of the arteriovenous network in adult stages as well as in neonatal stages.

### Supplementation with BMP10 but not BMP9 protein prevents AVM formation in Bmp9/10-deficient and ENG-deficient mice

**Fig. 7:**
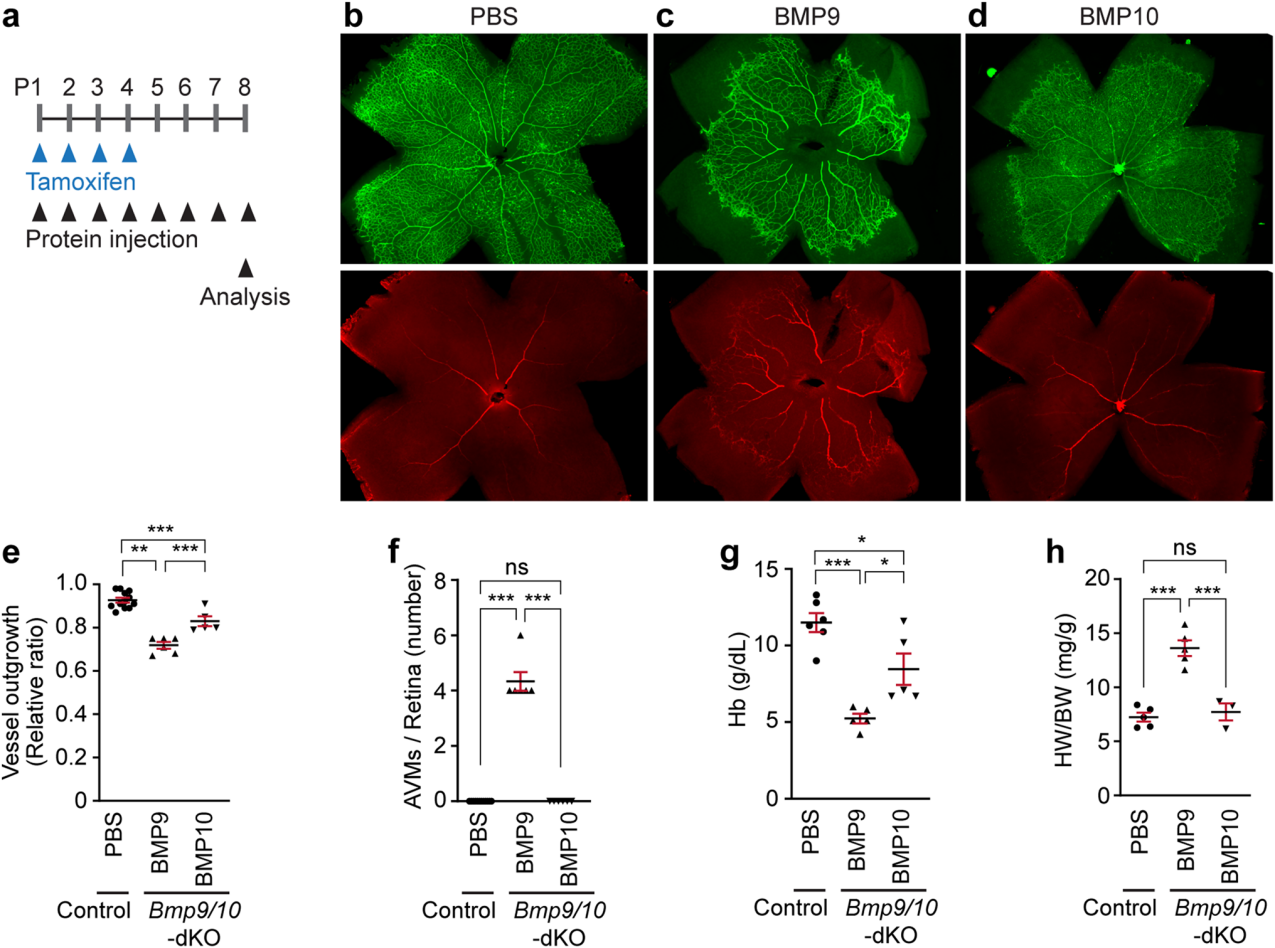
Administration of BMP10, but not BMP9, prevents AVM formation in *Bmp9/10*-dKO. **a** Schematic representation of BMP protein administration and *Bmp10* gene deletion into control and *Bmp9/10*-dKO mice. Arrowheads indicate injection of 50 μg tamoxifen at P1–P4 and BMP9 or BMP10 proteins daily from P1. **b–d** CD31 and SMA staining on retinas isolated from P8 control (PBS-treated CreER-negative *Bmp10*-iKO, **b**, BMP9-treated *Bmp9/10*-dKO (**c**), and BMP10-treated *Bmp9/10*-dKO (**d**) mice. Arrows mark arteriovenous shunts. *a* artery, *v* vein. Scale bars, 500 μm. **e** and **f** Quantification of retinal vascular outgrowth (**e**, *n* = 5 to 12 retinas from 5 to 6 mice per group) and AVM number (**f**, *n* = 6 to 12 retinas from 5 to 6 mice per group). Data are mean ± SEM. Statics Kruskal–Wallis test followed by Dunn’s post hoc test. **g** and **h** Hemoglobin levels (**g**, *n* = 5 to 6 mice per group) and HW/BW ratio (**h**, *n* = 3 to 5 mice per group) at the end day of the study. Data are mean ± SEM. One-way ANOVA followed by Tukey’s post hoc test

As shown in Figs. 1 and 2, *Bmp9/10*-dKO mice treated with tamoxifen for 4 days exhibit premature death by P7 and display severe vascular expansion in the retinal vasculature. We investigated whether systemic supplementation with BMP9 or BMP10 protein could prevent abnormal vascular development and premature death shown in *Bmp9/10*-dKO mice. Control (PBS), recombinant mouse BMP9, or BMP10 protein was injected daily until the sample collection, along with injections of 50 μg tamoxifen P1 through 4 (Fig. 7a). Supplementation with BMP9 or BMP10 protein protected *Bmp9/10*-dKO mice from premature death at P6 and enabled them to survive beyond P8. Retinal vasculature of PBS-treated *Bmp9/10*-dKO mice at P6 had the severe vascular expansion defects, similar to the images shown in Fig. 2b–e (not shown). We compared retinal vasculature of control (CreER-negative) treated with PBS with *Bmp9/10*-dKO mice treated with BMP9 or BMP10 proteins at P8. Compared to *Bmp9/10*-dKO retinas at P6 (Fig. 2b–e), which had no SMA-positive vessels and severe expansion defects, BMP9 or BMP10 treatment significantly increased vascular outgrowth, but not to the extent seen in PBS-treated control retinas (Fig. 7b–e). BMP10 was more effective than BMP9 on promoting the outgrowth (Fig. 7e). While CD31 and SMA stained *Bmp9/10*-dKO retinas treated with BMP10 (Fig. 7d) were almost indistinguishable from those of control retinas (Fig. 7b), those treated with BMP9 exhibited multiple abnormal SMA-positive veins connected to arterial branches (i.e., AV shunts) (Fig. 7c and f). Supplementation of BMP10 protein alleviated anemia and heart phenotypes (Fig. 7g and h).

**Fig. 8:**
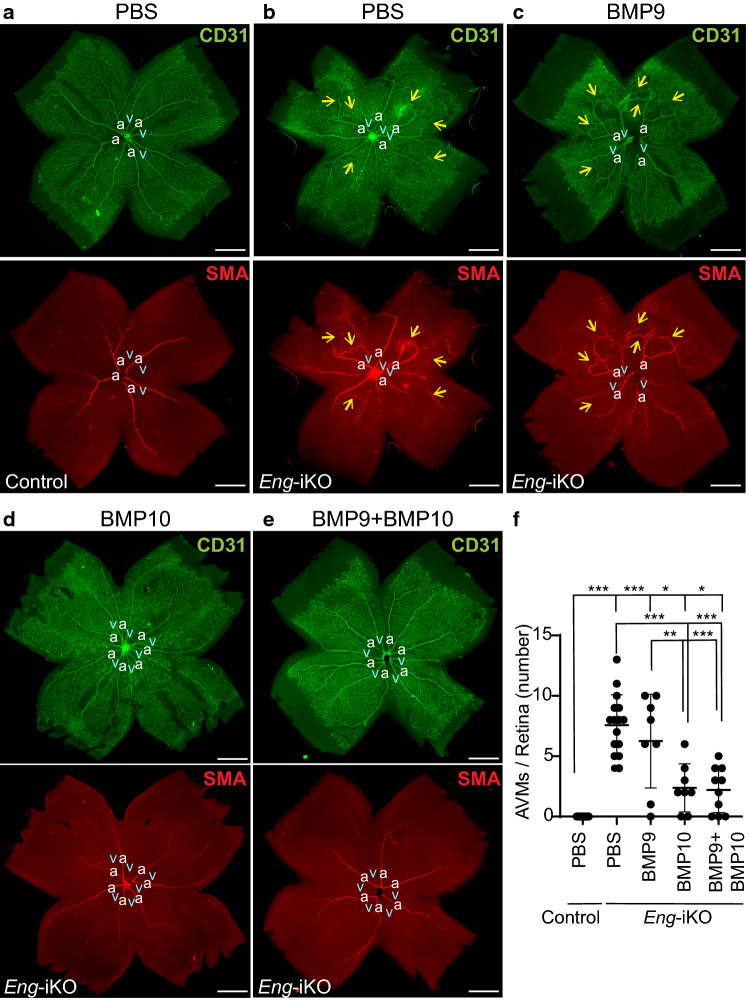
BMP10, but not BMP9, suppresses the development of AVMs caused by ENG-deficiency. **a**–**e** CD31 and SMA immunofluorescence staining on retinas isolated from P7 control (PBS-treated *Scl-*CreER-negative *Eng*-iKO, **a**, *n* = 28 retinas), PBS-treated *Scl-*CreER;*Eng*-iKO (**b**, *n* = 16), BMP9-treated *Scl-*CreER;*Eng*-iKO (**c**, *n* = 8), BMP10-treated *Scl-*CreER;*Eng*-iKO (**d**, *n* = 8), and BMP9/BMP10-treated *Scl-*CreER;*Eng*-iKO (**e**, *n* = 10) mice. Arrows mark arteriovenous shunts. *a* artery, *v* vein. Scale bars, 500 μm. **f** Quantification of the number of AVMs. Data are mean ± SD. One-way ANOVA followed by Tukey’s post hoc test

In order to validate this result and to assess the therapeutic potential of BMP9 or BMP10 in HHT, we performed the supplementation experiment on *Scl*-CreER( +);*Eng*^2f/2f^ (*Eng*-iKO) mice. We have previously shown that *Eng* deletion by an EC-specific *Scl*-CreER induced retinal AVM development [45]. The numbers of AVMs in the retinas of PBS-treated control and *Eng*-iKO mice (Fig. 8a, b) were compared with those of BMP9, BMP10, or both BMP9 and BMP10-treated *Eng*-iKO mice (Fig. 8c–e). The AVM numbers were significantly reduced in BMP10 and both BMP9 and BMP10-treatment groups, but not in the BMP9-treatment group (Fig. 8f). There was no difference between BMP10 and both BMP9/10-treatment groups.

## Discussion

HHT is a genetic vascular disease caused by mutations in *ENG*, *AKL1*, or *SMAD4* [4–6]. Identification of SMAD4 mutations in a subset of HHT patients having juvenile polyposis increased the possibility that HHT is a disease caused by defects in the signaling of a TGF-β family member(s) [6]. The report showing that endothelium-specific deletion of *Smad1* and *Smad5* resulted in premature death and retinal AVMs further supported important role of ENG-ALK1-SMAD signaling in the pathogenesis of HHT [46]. Finding a BMP9 locus by the linkage analysis to a family with HHT-like symptoms indicated that BMP9 is the physiological ligand of ENG-ALK1 signaling [7, 8]. Pathogenic mutations in the BMP9 binding site of ENG strengthened this notion [12, 13]. However, whether BMP9, its close subfamily member BMP10, or both function as a ligand(s) for ENG-ALK1 signaling pertinent to HHT pathogenesis has been unclear.

The importance of determining the ligand for ENG-ALK1 signaling is several fold. First, it could resolve further the issue to define HHT as a disease caused by the impairment of a linear signaling pathway. Second, it will be critical for in vitro studies to determine downstream mediators and targets of this signaling pathway. Cultured endothelial cells have been utilized to determine downstream targets of ENG-ALK1 signaling and cellular characteristics of ENG- or ALK-deficient cells. Including the proper ligand of this signaling in the EC culture media is pivotal for such assays. Third, it will also allow selecting the correct choice for a ligand-based therapy for HHT.

Conditional deletions of *Eng* [25, 39, 41], *Alk1* [24, 40], or *Smad4* [26, 27, 38] in mice exhibited vascular phenotypes closely mimicking vascular defects found in HHT patients. Although treatments with neutralizing antibodies for both BMP9 and BMP10 showed AVM phenotypes in the retinal vasculature, distinctive roles of each ligand could not be determined [20–23]. In addition, these antibody-based approaches were not adequate for assessment in adult mice. Genetic ablation experiments could be the clearest and most stringent test to resolve this issue. We demonstrate that *Bmp9/10*-dKO mice exhibited AVM phenotypes in the retina and brain of neonates and wound-induced skin AVMs at adult mice. Interestingly, *Bmp10*-iKO mice also developed AVMs similar to *Bmp9/10*-dKOs, while *Bmp9*-KO mice did not. This result indicates that BMP10 plays an independent role from BMP9 in the development of a proper arteriovenous network and is likely the physiological ligand for ENG-ALK1 signaling pertinent to HHT pathogenesis. This result is consistent with studies with zebrafish models in that double knockouts of *bmp10* and *bmp10-like* phenocopied AVMs phenotypes observed in *alk1*-null fishes [17]. On the other hand, this result contradicts the results from studies using independently generated *Bmp10*-iKO strain, in which no lethality or vascular malformation was reported [47]. Interestingly, Tillet et al. used the same targeting strategy with ours (i.e., targeting exon 2 of the *Bmp10* allele) and utilized R26^CreER^ mice for inducing gene deletion. The tamoxifen regimen in Tillet et al. (1 mg for 3-week-old mice for five consecutive day injection) was also comparable to the condition used in our study (0.1 mg/g body weight for three consecutive day injection). Recently, the same *Bmp10*-iKO mice was used to generate *Bmp9/10*-dKO mice in which decreased peripheral vascular resistance and signs of arteriovenous shunting were observed by the use of a bead assay, but morphological evidence of AVMs was not provided [30]. The reasons for the discrepancies between these two *Bmp10*-iKO strains are unknown. We speculate that the recombination efficiency may differ between these two conditional alleles. We demonstrate that the *Bmp10* locus was correctly targeted and judged by the lacZ expression in the reporter allele. Phenotypes of the null embryos (*Bmp10*^1f/1f^) and embryos in which *Bmp10* gene is conditionally deleted in cardiomyocytes were essentially identical with *Bmp10*^−/−^ embryos previously published by an independent group [15]. Our data also contradict to the hypothesis that the heterodimeric forms of BMP9:BMP10 but not the homodimers of BMP9 and BMP10 have physiological functions [47].

The AVM phenotypes observed in *Bmp10*-iKO or *Bmp9/10*-dKO appeared to be weaker than those in *Eng*, *Alk1*, or *Smad4-*iKO mice*.* Nevertheless, these AVMs exhibited the characteristics of AVM vessels shown in *Alk1*- [21, 22, 24, 45], *Eng*- [25, 37, 39, 45], or *Smad4* [26, 27, 38] models, such as abnormal SMA expression in veins, increased diameter of connecting vessels, and increased size and proliferation of endothelial cells. There are several possibilities for the weak appearance of AMVs in these genetic models. One is that the Cre-mediated recombination on the *Bmp10* allele is not complete. It has been shown that mosaic deletions of *Eng* or *Alk1* are sufficient for the development of AVMs, reflecting the cell-autonomous effect of deficiencies of the membrane or intracellular proteins [22, 37, 48]. In contrast to this, for secreted proteins like BMP10, mosaic deletion could result in a reduced level of protein, which could still be sufficient for its biological function. Another possibility might be related to the half-life of BMP10 in sera. Since *Bmp10*-iKO mice were lethal, there was a limited time window to observe vascular phenotypes. Although the BMP10 level in sera of *Bmp10*-iKO monitored by ELISA at 7 days after the first tamoxifen injection was almost undetectable, the levels of BMP10 on earlier days are still unknown. In addition, the tamoxifen injection regimen had to be changed because four consecutive days of injection starting from P1 resulted in too severe vascular phenotypes to observe AVM development. Two days of injection at P1/P3, P2/P3, or P2/P4 resulted in abnormal AV connections in *Bmp10*-iKOs or *Bmp9/10*-dKOs, but their phenotypes were much milder than those shown by BMP9/10 antibody treatment [20, 21, 23]. The neutralizing effects of antibody treatment could be instantaneous, but gene deletion only prevents the production of transcripts. BMP10 proteins and mRNAs produced prior to the gene deletion would be progressively depleted depending on their half-lives. AVMs in the retinas of *Bmp10*-iKO or *Bmp9/10*-dKO pups were detected in the distal areas of the retina, while AVMs developed in the proximal areas of BMP9/10 antibody-treated retinas [21–23] indicating the effects of BMP10 deficiency in antibody-treated conditions occur sooner than that in the gene deletion approaches.

It was reported that *Bmp9*-KO mice exhibit defects in the development of lymphatic vessels and valves [49, 50]. Despite no obvious vascular defect was detected in *Bmp9*-KOs, *Bmp9/10*-dKO showed aggravated phenotypes compared to *Bmp10*-iKOs. These phenotypes include lethality and vascular expansion in the retina, indicating that BMP9 compensates for the loss of BMP10. Nonetheless, there was an insignificant difference in AVM formation between *Bmp10*-iKO and *Bmp9/10*-dKO groups, indicating that BMP10 has a unique function, non-overlapping with BMP9 in the AV network formation. Consistent with this view, supplementation of BMP10 could prevent AVM development as well as vascular expansion defects, while BMP9 could inhibit vascular expansion defects but not AVM phenotype in *Bmp9/10*-dKO retinas. We further demonstrate that retinal AVMs in *Eng*-iKOs were inhibited by BMP10 supplementation. Together with our recent finding that over-expression of ALK1 could rescue ENG-deficiency [45], these results infer the therapeutic potential of BMP10 for HHT1 patients caused by ENG-deficiency.

Knowing that both ligands can signal through the same receptor complexes and effectively phosphorylate the same downstream SMAD proteins, it is enigmatic that BMP10 could but BMP9 could not prevent AVMs in *Bmp9/10*-dKO mice. One explanation is that there may be differences of active proteins in the plasma. We used intraperitoneal injection of the mature form of BMP9 and BMP10 homodimers with a high dosage of protein (100 ng) to observe the effects. Because *Bmp9* is completely depleted while *Bmp10* is gradually depleted, the plasma level of BMP10 is higher than that of BMP9 in *Bmp9/10*-dKO. Pharmacokinetics measurement in the supplementation study would provide the clues if the differences were derived from their functional differences or from their effective protein quantities. Our results also warrant further studies to decipher the differences between BMP9 and BMP10 for the biosynthesis, the mechanism of activation, half-lives, signaling mediators, and downstream target genes.

## Supplementary Information

Below is the link to the electronic supplementary material.

Supplementary file1 (PDF 4083 kb)

Supplementary file2 (PDF 90 kb)

## Abbreviations

AV: Arteriovenous
AVM: Arteriovenous malformation
BABB: Benzyl alcohol: benzyl benzoate
BBB: Blood–brain barrier
BMP: Bone morphogenetic protein
DiceCT: Diffusible iodine-based contrast-enhanced computed tomography
ECs: Endothelial cells
Hb: Hemoglobin
HHT: Hereditary hemorrhagic telangiectasia
E: Embryonic day
P: Postnatal day
iKO: Inducible knockout
dKO: Double knockout
TGF-β: Transforming growth factor-β
WT: Wild-type
