# Supplementary material for: BMP10 functions independently from BMP9 for the development of a proper arteriovenous network
Source: Angiogenesis. 2022 Nov 8;26(1):167–86. doi: 10.1007/s10456-022-09859-0 (PMC9908740; doi:10.1007/s10456-022-09859-0)
Supplement: Supplementary file 1 — Supplementary file1 (PDF 4083 kb) [file 10456_2022_9859_MOESM1_ESM.pdf]

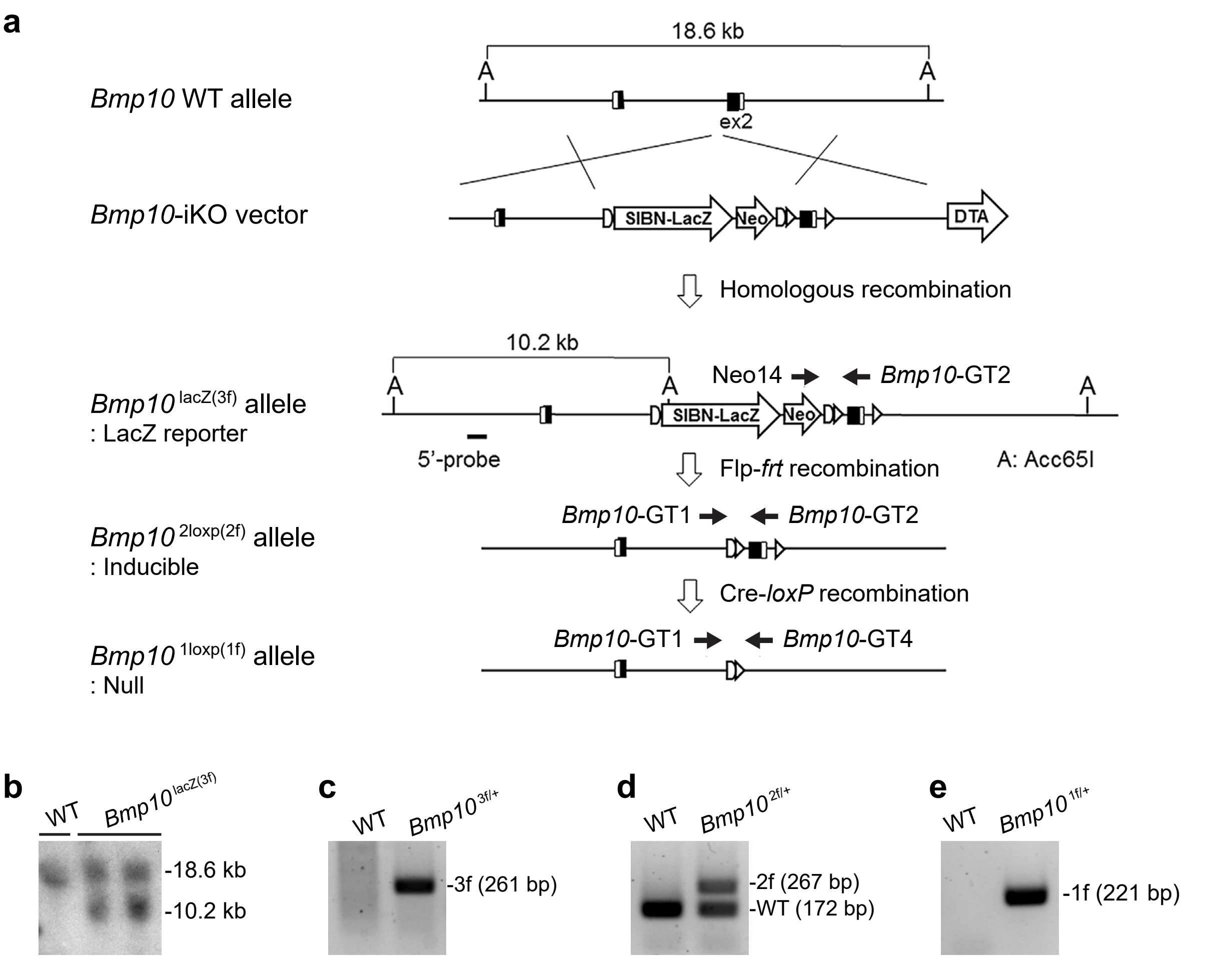

Figure S1: Generation of Bmp10-reporter, conditional, and null alleles.
a, Schematic diagram of *Bmp10* wild-type (WT), lacZ reporter (*Bmp10*$^{lacZ(3f)}$), conditional (*Bmp10*$^{2loxP(2f)}$), and null (*Bmp10*$^{1loxP(1f)}$) alleles. The targeting vector consists of a 5.6 kb 5'-homology arm, a 4.0 kb 3'-homology arm, a frt-SD/SA-IRES-LacZ-Neo-frt-loxP (LacZ/Neo) cassette (SIBN: SA, splice donor and acceptor; IRES, internal ribosome entry sequence; LacZ, -galactosidase gene; Neo, neomycin resistance gene), and a DTA (Diphtheria toxin A fragment gene) cassette. Exons are represented by boxes. The coding region is indicated by black boxes. Frt sequences flanking the LacZ/Neo cassette and loxP sequences flanking exon2 are indicated by semicircles and triangles, respectively. The 5' probe for Southern hybridization is shown in the WT alleles. Locations of primer pairs used for genotyping are indicated by arrows. **b,** Southern blot analyses of selected ES cells. Genomic DNAs isolated from selected ES clones were digested with Acc65I and then hybridized with the 5'-probe. The probe detected 18.6-kb and 10.2 kb fragments for the *Bmp10* ^{WT} and *Bmp10*^{3f} alleles, respectively. **c-e,** Genotyping of heterozygous alleles of 3f (**c**), 2f (**d**), and 1f (**e**) with specific primer pairs shown in panel a.

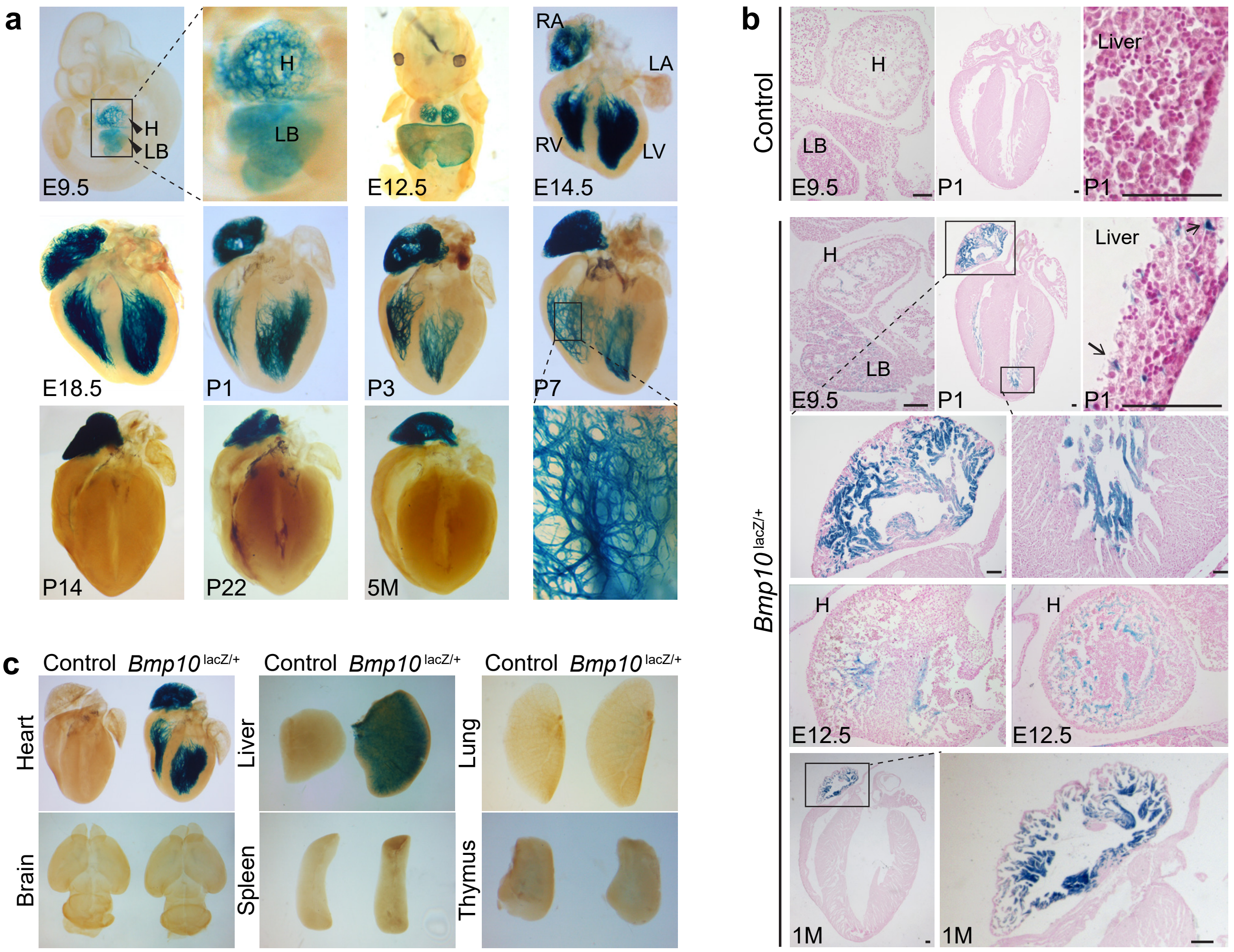

Figure S2: BMP10 is expressed in the ventricles and right atrium of neonatal hearts.
a, X-gal staining of *Bmp10*$^{lacZ/+}$ embryos and hearts to visualize Bmp10 expression in the indicated embryonic, postnatal, and adult stages. E, embryonic day; P, postnatal day; M, month-old; H, heart; LB, liver bud; L, liver; RA, right atrium; LA, left atrium; RV, right ventricle; LV, left ventricle. **b**, Nuclear Fast Red (NFR) stained sections of whole-mount X-gal-stained heart and liver from control and *Bmp10*$^{lacZ/+}$ mice. Scale bar, 100µm. **c,** X-gal staining of various organs from control and *Bmp10*$^{lacZ/+}$ at P1.

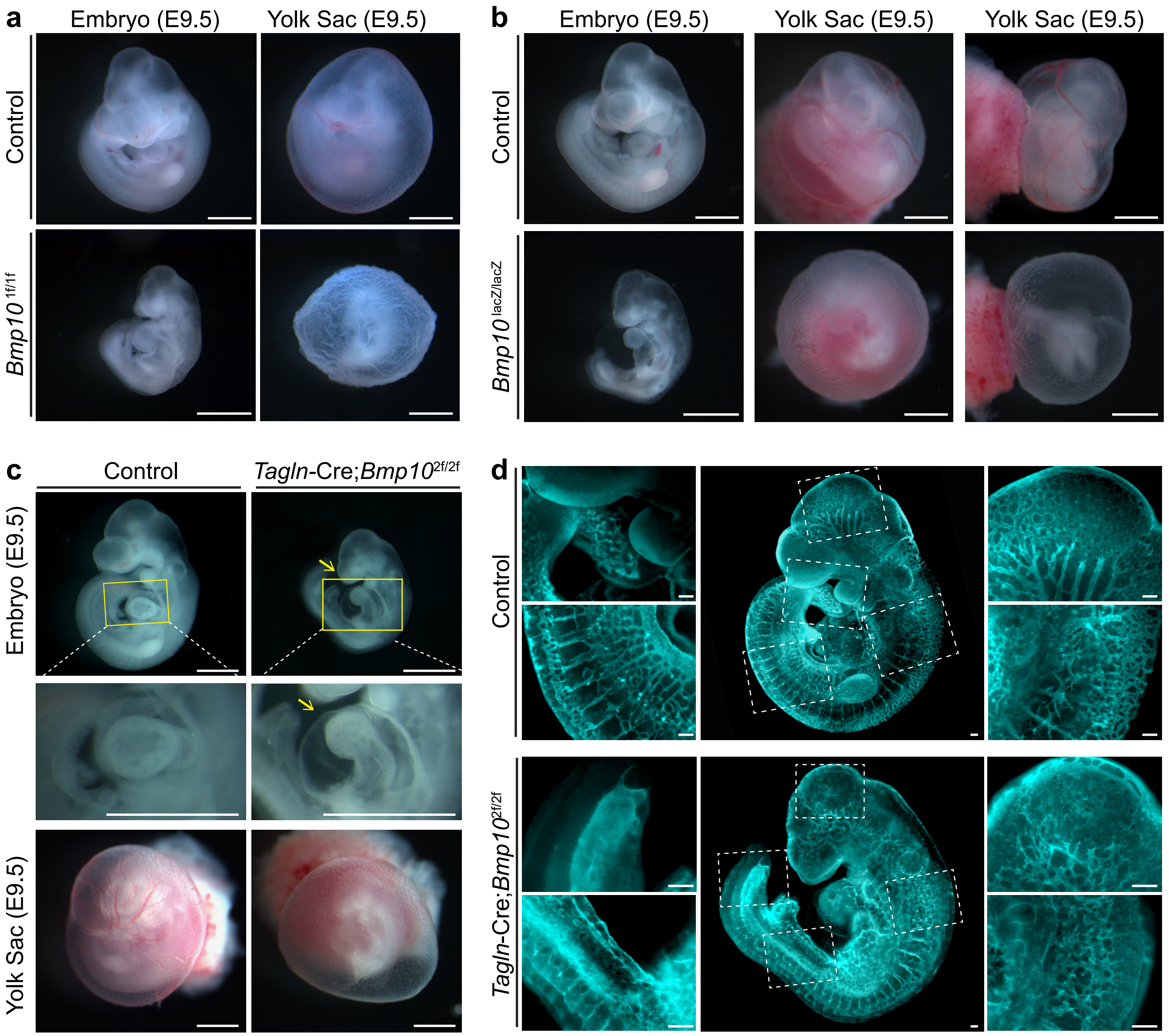

Figure S3: Embryonic lethality of mice with *Bmp10*-null alleles.
a-c, Gross morphology of embryos and yolk sacs from littermate control and *Bmp10*$^{1f/1f}$ (**a**), *Bmp10*$^{lacZ/lacZ}$ (**b**), and *Tagln*-Cre;*Bmp10*$^{2f/2f}$ (**c**) at E9.5. Arrows mark prominent pericardial effusions. Scale bars, 1mm. d Whole-mount immunohistochemical analysis of control and *Tagln*-Cre;*Bmp10*$^{2f/2f}$ E9.5 embryos with anti-CD31 antibodies. Insets indicate the magnified area. Scale bars, 100 μm.

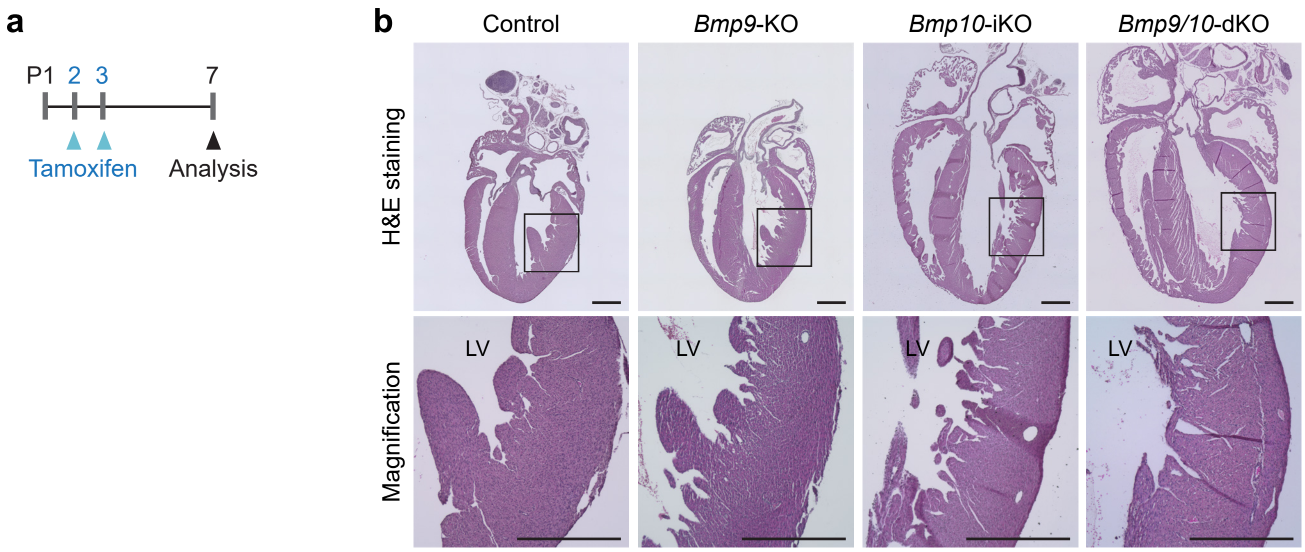

Figure S4: Bmp10-deleted neonates exhibit cardiac phenotypes.
a, Schematic representation of *Bmp10* deletion in neonatal mice. Blue arrowheads indicate the days of intragastric injections of tamoxifen at P2 and P3. **b,** H&E staining of heart sections from P7 control, *Bmp9*-KO, *Bmp10*-iKO, and *Bmp9/10*-dKO mice. Insets indicate the magnified area. Scale bars, 500 µm. LV, left ventricle.

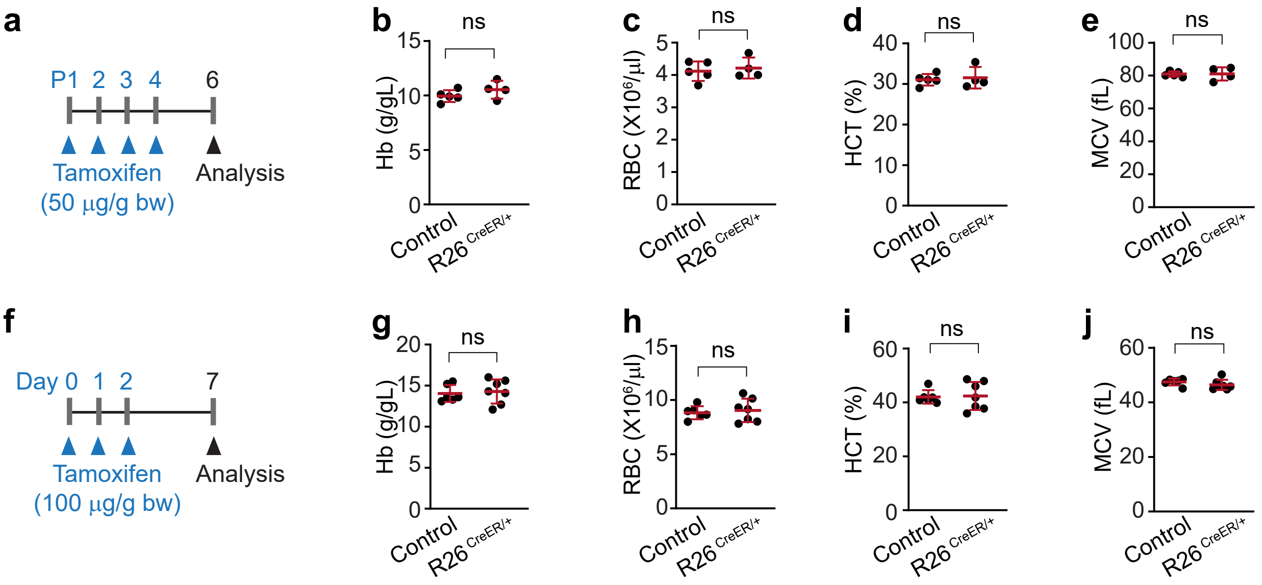

Figure S5: CreER activation with tamoxifen injection windows used for *Bmp10* gene deletion does not change anemia-related blood counts.
a, Schematic representation of the experimental strategy for CreER activation in neonatal control (CreER-negative $Bmp10^{+/+}$ or $Bmp10^{2f/+}$) and $R26^{CreER/+}$ (CreER-positive $Bmp10^{+/+}$) mice from the mating of $R26^{CreER/+}$;$Bmp10^{2f/+}$ and $Bmp10^{+/+}$ mice. The arrowheads indicate the days of intragastric injection of 50 µg tamoxifen for consecutive 4 days from postnatal day 1 (P1). Complete blood counts were carried out on P6. **b-e,** Scatter plots measuring hemoglobin level (Hb, **b**), red blood cell count (RBC, **c**), hematocrit (HCT, **d**), and mean corpuscle volume (MCV, **e**) to assess anemia. Data are mean ± SD (n = 4 to 5 mice per group). Unpaired two-tailed student's *t*-test. **f,** Schematic representation of the experimental strategy for CreER activation in adult control (CreER-negative $Bmp10^{+/+}$ or $Bmp10^{2f/+}$) and $R26^{CreER/+}$ (CreER-positive $Bmp10^{+/+}$) mice. CreER activation was induced by intraperitoneal injection of tamoxifen at 100 µg/g body weight for 3 consecutive days from day 0, and then complete blood counts were carried out at day 7. **g-j,** Scatter plots measuring hemoglobin level (Hb, **g**), red blood cell count (RBC, **h**), hematocrit (HCT, **i**), and mean corpuscle volume (MCV, **j**) to assess anemia. Data are mean ± SD (n = 7 to 8 mice per group). Unpaired two-tailed student's *t*-test.

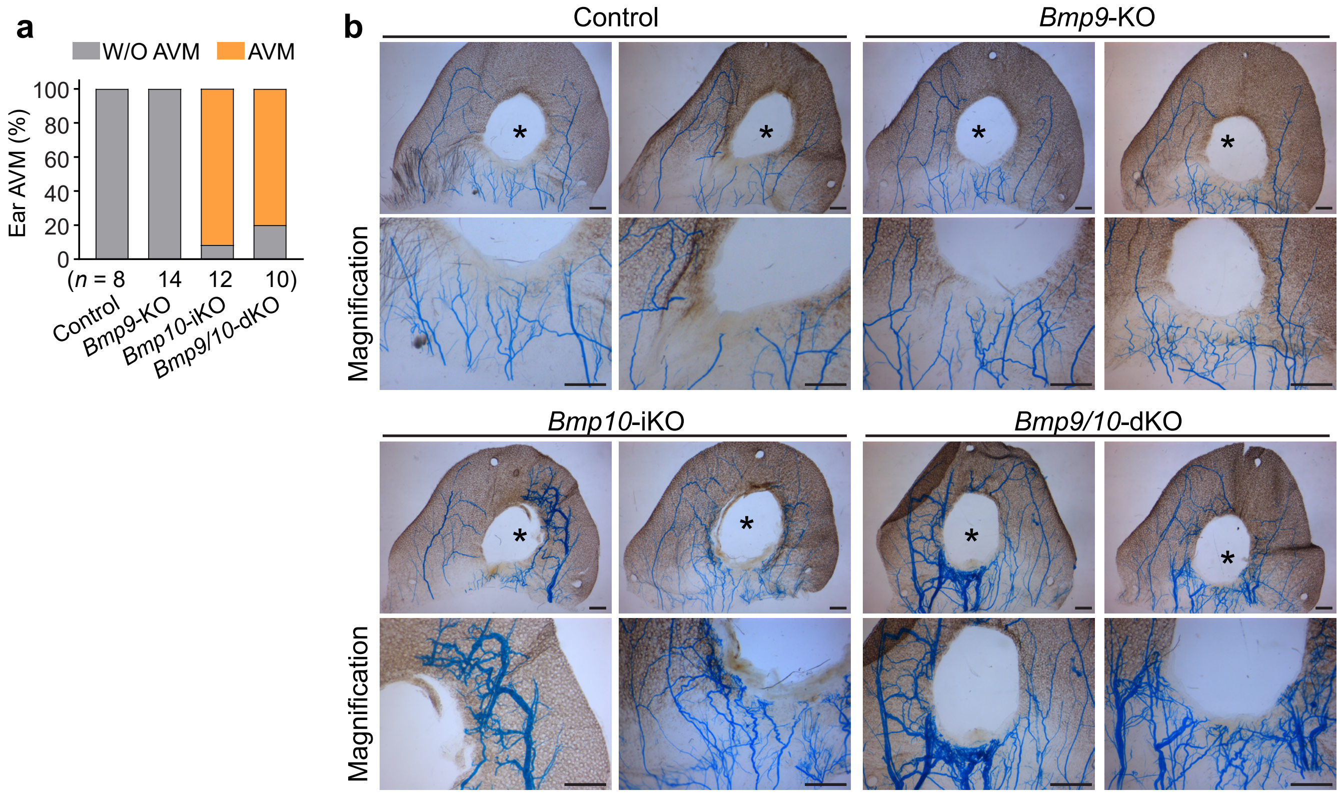

Figure S6: *Bmp10* deletion leads to AVM in ears in response to wounding in adults.
a, Representative images of latex dye-injected blood vessels on wounded ears of control, *Bmp9*-KO, *Bmp10*-iKO, and *Bmp9/10*-dKO mice. The wound sites are indicated by asterisks. Scale bars, 1 mm.
b, Percentage of mice having ear wound-induced AVM (n = 8 to 14 mice per group).
