# Supplementary material for: BMP10 functions independently from BMP9 for the development of a proper arteriovenous network
Source: Angiogenesis. 2022 Nov 8;26(1):167–86. doi: 10.1007/s10456-022-09859-0 (PMC9908740; doi:10.1007/s10456-022-09859-0)
Supplement: Supplementary file 2 — Supplementary file2 (PDF 90 kb) [file 10456_2022_9859_MOESM2_ESM.pdf]

Online Table I. The list of genotyping primers

Target	Primer Name	Sequence (5´-3´)
Bmp10^{1f}	*Bmp10*-GT1	GGC TCA TCT ACA GCT CCA AGA TTC
	Bmp10-GT4	GTG CAC CTG CAT GAA CGT GCA CAA
Bmp10^{2f}	*Bmp10*-GT1	GGC TCA TCT ACA GCT CCA AGA TTC
	Bmp10-GT2	TTG ATG ATA ATG GAC TGA ACC TCT
Bmp10$^{lacZ(3f)}$	Neo14	CTC TAT GGC TTC TGA GGC GGA AAG
	Bmp10-GT2	TTG ATG ATA ATG GAC TGA ACC TCT
Bmp9	*Bmp9*-FW	GAA GTA TCG AGT GCC GTG AAG CGG T
	Bmp9-Re-WT	CTC CTG CTC ATG GCC GAT CAT CTC C
	Bmp9-Re-KO	CGC CTT CTT GAC GAG TTC TTC TGA GG
CreER	CreER-F	CAT GAA CTA TAT CCG TAA CCT GGA
	CreER-R	CAT CCA ACA AGG CAC TGA CCA TCT
Tagln-Cre	Cre-F	GCG GTC TGG CAG TAA AAA CTA TC
	Cre-R	GTG AAA CAG CAT TGC TGT CAC TT
